# Genomic and immunological differences in endometrial cancer: a comparative study between young and old Asian patients

**DOI:** 10.3389/fimmu.2026.1737794

**Published:** 2026-02-10

**Authors:** Senwei Jiang, Yunhui Li, Shanshan Wu, Jing Wan, Xiaomao Li

**Affiliations:** Department of Gynecology, The Third Affiliated Hospital of Sun Yat-sen University, Guangzhou, China

**Keywords:** age-related genomics, endometrial cancer, Asian, mutation, molecular mechanisms, MSI, TMB

## Abstract

**Background:**

Endometrial carcinoma (EC) demonstrates a pronounced age-related disparity in clinical outcomes, yet the underlying genomic and molecular mechanisms remain incompletely characterized, especially across Asian populations.

**Methods:**

We performed an integrated genomic analysis of two independent cohorts: the MSK-MET Asian cohort (n=94) and a Chinese EC cohort, EC-3rd-SYSU (n=18). Patients were stratified by age (young, ≤50 years; old, >50 years). We compared mutation spectra, pathway enrichment, tumor mutation burden (TMB), microsatellite instability (MSI), and survival outcomes between age groups.

**Results:**

Across both cohorts, PTEN, PIK3CA, and ARID1A were the most frequently mutated genes, forming a conserved oncogenic core centered on PI3K/AKT signaling and chromatin remodeling. Younger patients showed enrichment of CTNNB1 and PTEN mutations, while older patients exhibited higher frequencies of TP53, ARID1A, and MSH6 alterations. Younger tumors were characterized by activation of stemness and metabolic signaling pathways, whereas older tumors were enriched in cellular senescence, endocrine resistance, and longevity regulation pathways. No significant age-associated differences were observed in TMB or MSI status. The Chinese cohort had higher mutation rates of PTEN, KRAS, and FGFR2, while TP53 and PIK3R1 mutations were more frequent in the MSK-MET cohort. These age-specific molecular subtypes correlated with significant survival differences.

**Conclusions:**

Our study identifies conserved age-specific molecular subtypes of EC and reveals population-specific genomic features, underscoring the need for age-tailored and ethnicity-aware therapeutic strategies.

## 1 Introduction

Endometrial cancer (EC), or uterine corpus cancer, is the most common malignancy of the female reproductive tract in developed countries, and its incidence is increasing globally (1). In China, the rising incidence of EC is a cause for concern, particularly given the demographic shift towards an aging population (2). Traditionally, endometrial cancer is considered a disease of postmenopausal women, with the majority of cases occurring in women aged 60 years or older (3). However, there has been a disturbing trend of younger women, often under the age of 50, being diagnosed with this disease. This shift has led to the hypothesis that there may be unique molecular and genetic underpinnings in young patients with EC that differ from those in older women.

As such, age is often used as a clinical stratification variable in EC research and management. Numerous studies have adopted 50 years as the cut-off point to distinguish between “younger” and “older” patient populations. This threshold is supported by demographic data and clinical patterns, with 50 years commonly marking the menopausal transition, which brings about hormonal and metabolic changes that affect tumor behavior and estrogen responsiveness (4). It is reported that the median age of EC diagnosis in Chinese women is approximately 50 years, with a significant shift toward postmenopausal status and increased incidence of high-risk features beyond this age (5). Furthermore, survival analyses stratified by an age cutoff of 50 years have demonstrated significantly different prognostic outcomes among female cancer patients (6). Therefore, the use of 50 years as a stratification point is both epidemiologically justified and clinically meaningful.

Epidemiological studies have consistently shown that the incidence of EC increases with age, with the majority of cases diagnosed in postmenopausal women (7). Younger patients are more likely to present with early-stage, low-grade tumors and favorable histological subtypes such as endometrioid adenocarcinoma (8). In contrast, older women are at greater risk for had a higher frequency of deep myometrial invasion, serous tumor histology, and poorer clinical outcomes (9).

Immunotherapy, including FDA-approved pembrolizumab for advanced or recurrent MSI-H/dMMR endometrial cancer, has shown promising efficacy in improving prognosis, especially in cases with high tumor mutational burden (TMB) or microsatellite instability-high (MSI-H) status. These biomarkers are currently the primary criteria for selecting candidates for immunotherapy. Recent studies have also indicated a strong correlation between patient age and both TMB and MSI status (10). Older patients tend to have higher TMB and a greater likelihood of MSI-H tumors, which may influence their responsiveness to immune checkpoint inhibitors (11). Therefore, age should be considered alongside molecular markers when evaluating immunotherapy options for endometrial cancer patients.

While significant progress has been made in understanding the genetic landscape of EC, there is still a lack of comprehensive studies that examine the genomic differences between younger and older patients with EC, particularly in the context of Asian populations. This study aims to address this gap by performing a detailed genomic comparison between young (≤50 years) and elderly (>50 years) Asian patients with endometrial cancer. We hypothesize that the genomic alterations in these two groups differ significantly, and that these differences may contribute to the distinct clinical outcomes observed in these patients. We also aim to identify potential biomarkers and therapeutic targets that could lead to more personalized treatment strategies for EC.

## 2 Materials and methods

### 2.1 Study population

MSK-MET (Memorial Sloan Kettering - Metastatic Events and Tropisms) is a pan-cancer cohort of tumor genomic and clinical outcome data from 25,000 patients (12). The dataset identifies associations between tumor genomic alterations and patterns of metastatic dissemination across 50 tumor types; This study retrospectively included 94 Asian-far east/indian subcont patients diagnosed with endometrial cancer in MSK-MET. Except for two patients with missing age data, the remaining patients were divided into two groups based on age: young group (age ≤50 years, 19 patients) and elderly group (age>50 years, 73 patients). Clinical data, including age, histological subtype, metastatic site, mutation count, MSI, and TMB, were obtained from patient records.

The EC-3rd-SYSU (Endometrial cancer - Third Affiliated Hospital of Sun Yat-sen University) cohort, comprising 18 Chinese endometrial cancer patients retrospectively enrolled at the Third Affiliated Hospital of Sun Yat-sen University (2022-2024), underwent next-generation sequencing (NGS). Patients were excluded due to: unavailable matched normal tissue; low sequencing coverage (<100x); low tumor purity (evidenced by absence of somatic alterations, including silent mutations); age <18 years; multiple unique sequenced tumor types. The patients were divided into two groups based on age: young group (age ≤50 years, 10 patients) and elderly group (age>50 years, 8 patients). Clinical data, including age, histological subtype, metastatic site, mutation count, MSI, and TMB, were obtained from patient records.

### 2.2 Genomic analysis

The sequencing methodology and genomic analysis for the MSK-MET cohort are described in detail in the referenced publication (12). The genomic alterations were analyzed and download using cBioPortal (http://www.cbioportal.org).

For EC-3rd-SYSU cohort matched germline (blood) and tumor DNA samples were processed for targeted NGS using a customized gene panel (90 genes, **Supplementary Table S1**), which covered exonic regions, known hotspots, and selected intronic/promoter sequences. Sequencing was performed on an Illumina platform with depth-of-coverage thresholds set at ≥500x for tumor DNA and ≥100x for germline DNA to enable somatic variant calling.

Somatic Mutations: Somatic mutations were interpreted according to the Association for Molecular Pathology (AMP), the American Society of Clinical Oncology (ASCO), and the College of American Pathologists (CAP) guidelines. Only Tier I (strong clinical significance) and Tier II (potential clinical significance) variants are reported here. Somatic mutations were detected using MuTect2, and the variant calls were annotated using ANNOVAR. We focused on point mutations and small insertions/deletions (indels) in known cancer-related genes.

Pathway Enrichment Analysis: Kyoto Encyclopedia of Genes and Genomes (KEGG) analyses were performed to identify biological pathways significantly altered in young vs. elderly patients.

Tumor Mutational Burden (TMB) and Microsatellite Instability (MSI): TMB was calculated as the number of somatic, coding, nonsynonymous mutations per megabase of the panel’s targeted region. A tumor mutational burden (TMB) of ≥10 mutations per megabase (mut/Mb) was classified as high. MSI status was assessed using the NGS data from the tumor and matched normal samples. The sample was classified as MSI-High(MSI-H) if MSIsensor score >10, MSI-Low (MSI-L)/Microsatellite Stable (MSS) if MSIsensor score ≤10 as previously described (12).

### 2.3 Statistical analysis

The data was analyzed using SPSS (version 19.0) and cBioPortal. Statistical comparisons between the two groups were performed using the Chi-square test for categorical variables and the Student’s t-test for continuous variables. Differentially expressed genes were considered significant if they had a false discovery rate (FDR) < 0.05. A p-value < 0.05 was considered statistically significant for all other tests. Data visualization was performed with PowerPoint and Sangerbox tools (13).

## 3 Results

### 3.1 Genomic profile of Asian EC in MSK-MET cohort

**Figure 1:**
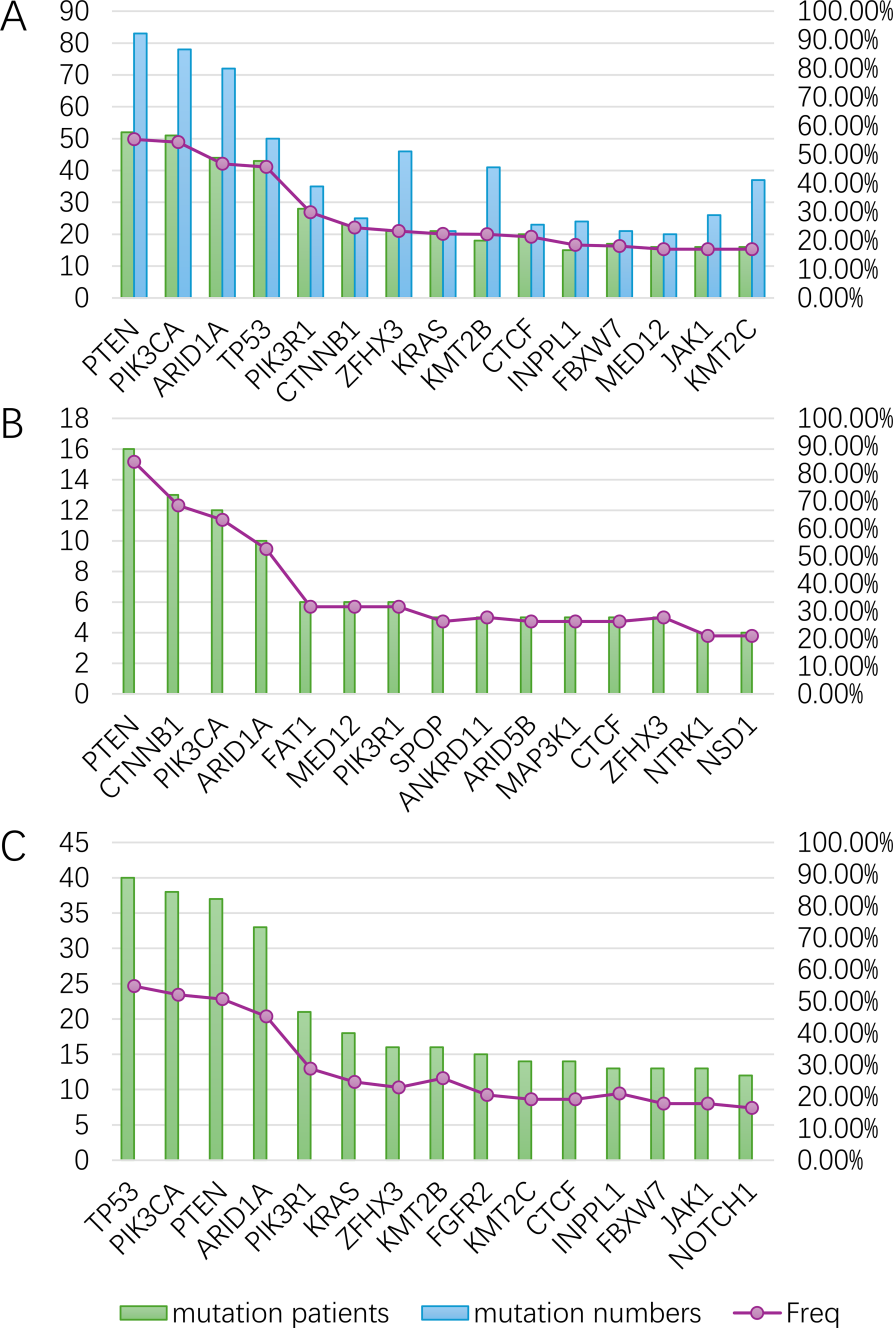
**(A)** Landscape of genomic alterations among asian EC patients in MSK-MET. The comparative analysis of frequencies of gene mutations between young **(B)** and old **(C)** groups with EC.

A total of 35,000 somatic mutations were identified across all samples. Samples from all 94 Asian patients were profiled by targeted sequencing, and all exhibited at least one genetic alteration. The most frequently mutated 5 genes were PTEN (55.3%; total number of mutations, n=83; number of samples with one or more mutations, n=52), PIK3CA (54.3%; n=78; n=51), ARID1A (46.8%; n=72; n=44), TP53 (45.7%; n=50; n=43), and PIK3R1 (29.8%; n=35; n=28). These were followed by genes ranked 6-15 — including CTNNB1 (24.5%), ZFHX3 (22.3%), KRAS (22.3%) and others — with mutation frequencies ranging from 24.5% to 16.0%.” (**Figure 1A**).

### 3.2 The clinical characteristics of Asian EC in MSK-MET cohort

After excluding two patients with missing age information, all remaining patients were divided into two groups based on age: young group (age ≤50 years, 19 patients) and old group (age>50 years, 73 patients). The clinical characteristics of Asian endometrial cancer patients in the MSK-MET cohort were compared between younger (n = 19) and older (n = 73) groups. A significant age difference was observed, with means of 39.93 ± 5.95 years in the younger group and 62.67 ± 7.51years in the older group (p = 2.25E-11). The distribution of histological subtypes differed significantly between groups (p = 4.04E-03), with all younger patients presenting with endometrioid carcinoma, while the older group included cases of serous carcinoma (n = 17) and carcinosarcoma (n = 12). No significant difference in metastasis rate (p = 0.052) and mortality (p = 0.140) were observed (**Table 1**).

**Table 1:** Clinical characteristics of asian EC patients in MSK-MET cohort.

Characteristics	Young (n = 19)	Old (n = 73)	p value
Age (mean,SD)	40.43±5.99	62.67±7.51	2.25E-11
Histological subtype			4.04E-03
Endometrioid Carcinoma	19	44	
Serous Carcinoma/Papillary Serous Carcinoma	0	17	
Carcinosarcoma/Malignant Mixed Mullerian Tumor	0	12	
Metastasis			0.052
yes	9	54	
no	10	19	
Death Status			0.140
Alive	17	52	
Dead	2	21	
MSI Type			0.641
Stable	16	57	
Instable	3	10	
Indeterminate	0	5	
Do not report	0	1	
MSI Score (mean,SD)	4.86±11.04	4.14±8.40	0.180
TMB (mean,SD)	30.00±49.65	31.86±90.27	0.324

### 3.3 Differences in mutation spectrums between young and old in MSK-MET cohort

Among young patients, the most frequently mutated 5 genes were PTEN (84.21%; number of samples with one or more mutations, n=16), CTNNB1 (68.42%, n=13), PIK3CA (63.16%, n=12), ARID1A (52.63%, n=10) and FAT1 (31.58%, n=6) (**Figure 1B**; **Supplementary Table S2**). In elderly patients, mutations in genes such as TP53 (54.79%, number of samples with one or more mutations, n=40), PIK3CA (52.05%, n=38), PTEN (50.68%, n=37), ARID1A (45.21%, n=33) and PIK3R1 (28.77%, n=21) (**Figure 1C**) were more prevalent.

**Figure 2:**
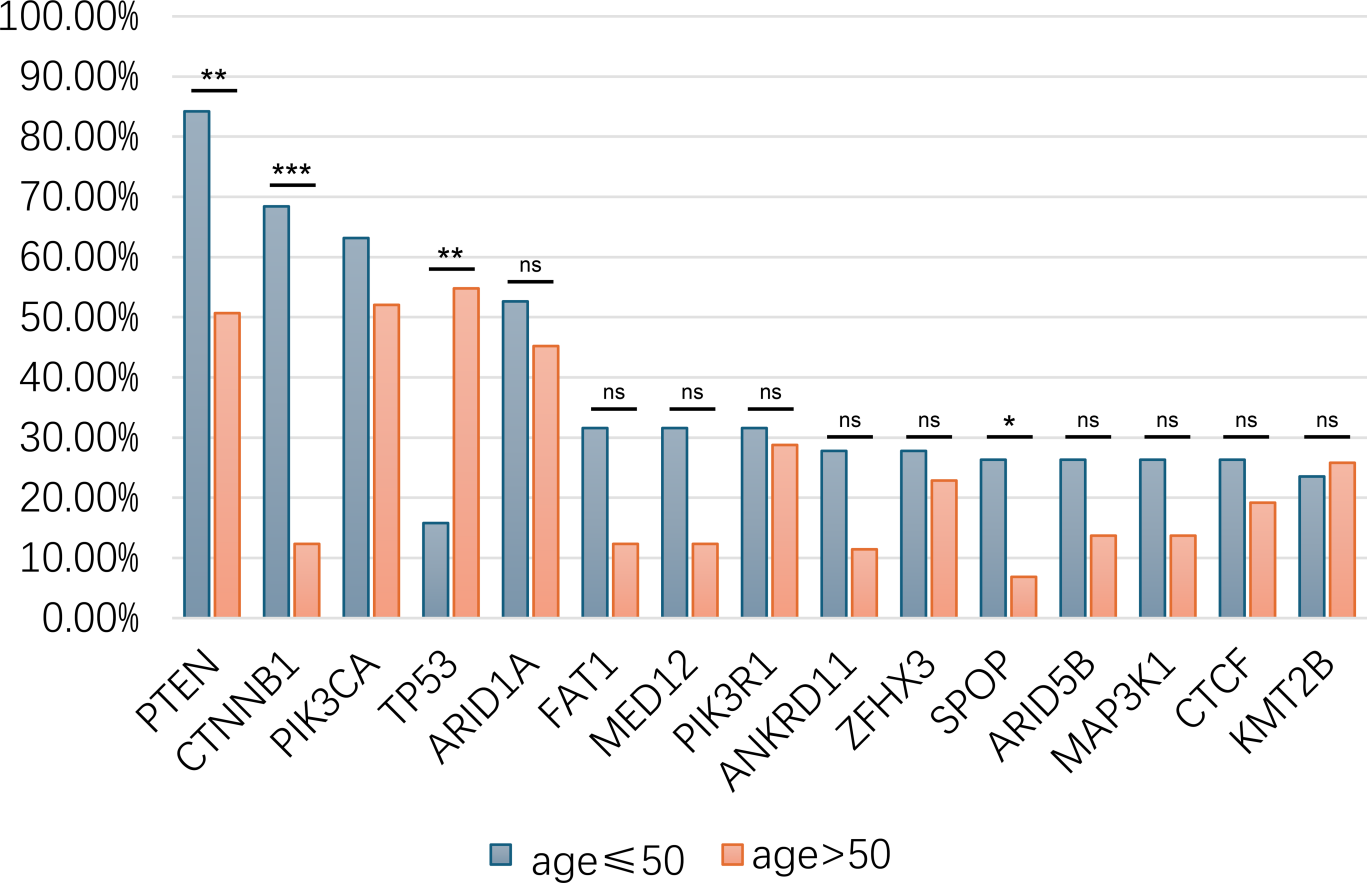
Genetic mutations difference between young and old patients in asian EC patients in MSK-MET. ns, not significantly; *, p <0.05; **, p <0.01; ***, p < 0.001.

Statistical analysis revealed that the frequencies of *CTNNB1* and *PTEN* mutations in the young group were significantly higher compared with that in the old group (68.42% vs 12.33%, *p* = 3.03e-6 and 84.21% vs 50.68% *p* = 2.709e-4, respectively), while *TP53* alterations were potentially associated with old age (54.79% vs 15.79%, *p* = 1.156e-4) (**Figure 2**).

### 3.4 Pathways and molecular mechanisms in MSK-MET cohort

To investigate the biological implications of age-related genomic differences in EC, we analyzed the 15 most frequently mutated genes (**Figures 1B, C**), followed by KEGG pathway enrichment analysis for each group.

**Figure 3:**
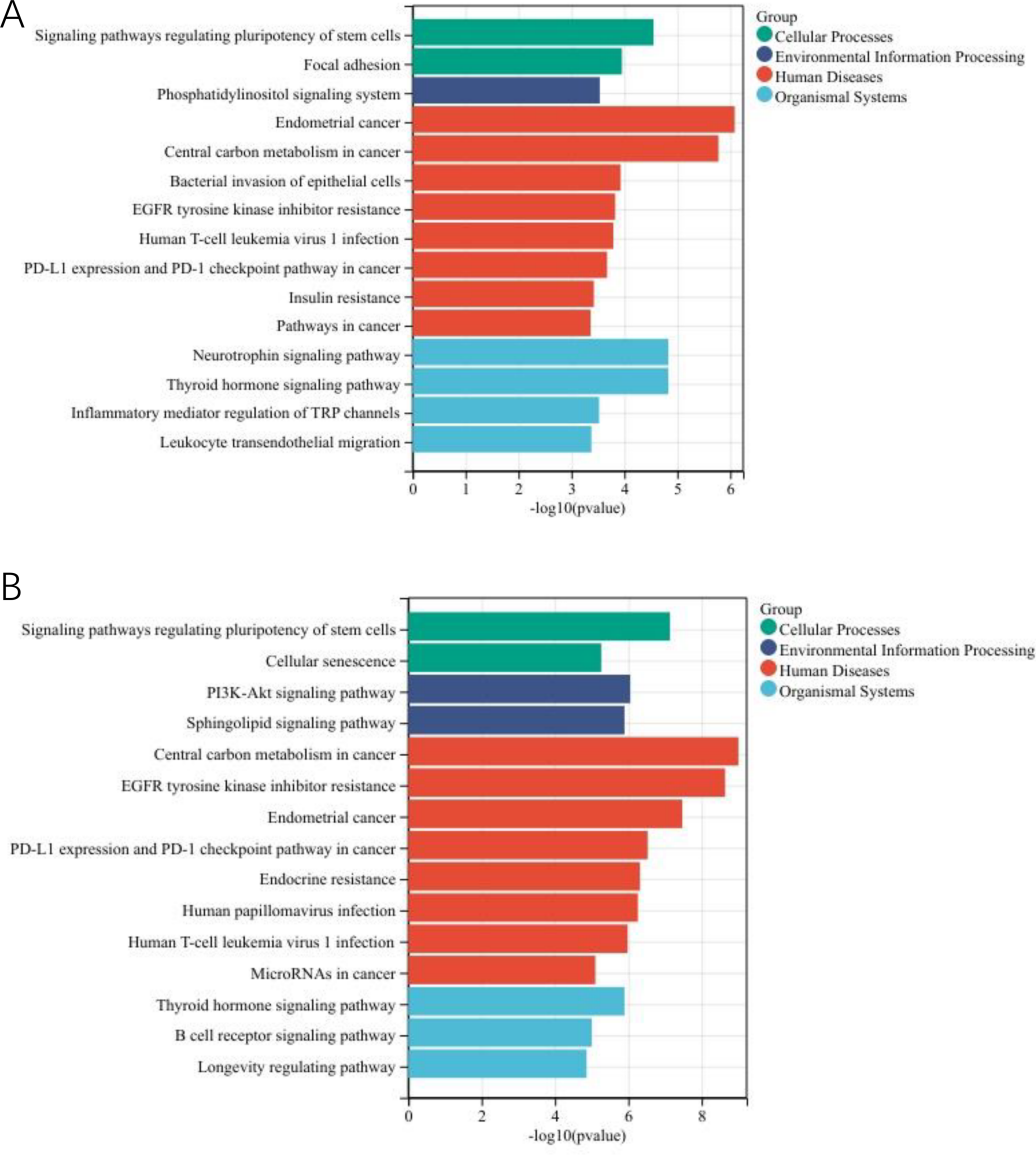
The most enriched KEGG pathways associated with actionable mutations in asian EC patients in MSK-MET. **(A)** The enriched pathways in the young group. **(B)** The enriched pathways in the old group.

Enrichment analysis revealed significant terms across multiple categories in young group: for cellular processes, signaling pathways regulating pluripotency of stem cells and focal adhesion; for environmental information processing, phosphatidylinositol signaling system; for human diseases, endometrial cancer, central carbon metabolism in cancer, bacterial invasion of epithelial cells, EGFR tyrosine kinase inhibitor resistance, Human T-cell leukemia virus 1 infection, PD-L1 expression and PD-1 checkpoint pathway in cancer and Insulin resistance; and for organismal systems, neurotrophin signaling pathway, thyroid hormone signaling pathway, inflammatory mediator regulation of TRP channels and Leukocyte transendothelial migration (**Figure 3A**).

In old group, cellular processes were enriched for signaling pathways regulating pluripotency of stem cells and cellular senescence. Environmental information processing were enriched for PI3K-Akt signaling pathway and sphingolipid signaling pathway. Human diseases featured central carbon metabolism in cancer, EGFR tyrosine kinase inhibitor resistance, endometrial cancer, PD-L1 expression and PD-1 checkpoint pathway in cancer, endocrine resistance, human papillomavirus infection, human T-cell leukemia virus 1 infection and MicroRNAs in cancer. These processes were anchored in organismal systems including thyroid hormone signaling pathway, B cell receptor signaling pathway and longevity regulating pathway (**Figure 3B**).

### 3.5 Age-stratified genomic instability correlates in MSK-MET cohort

**Figure 4:**
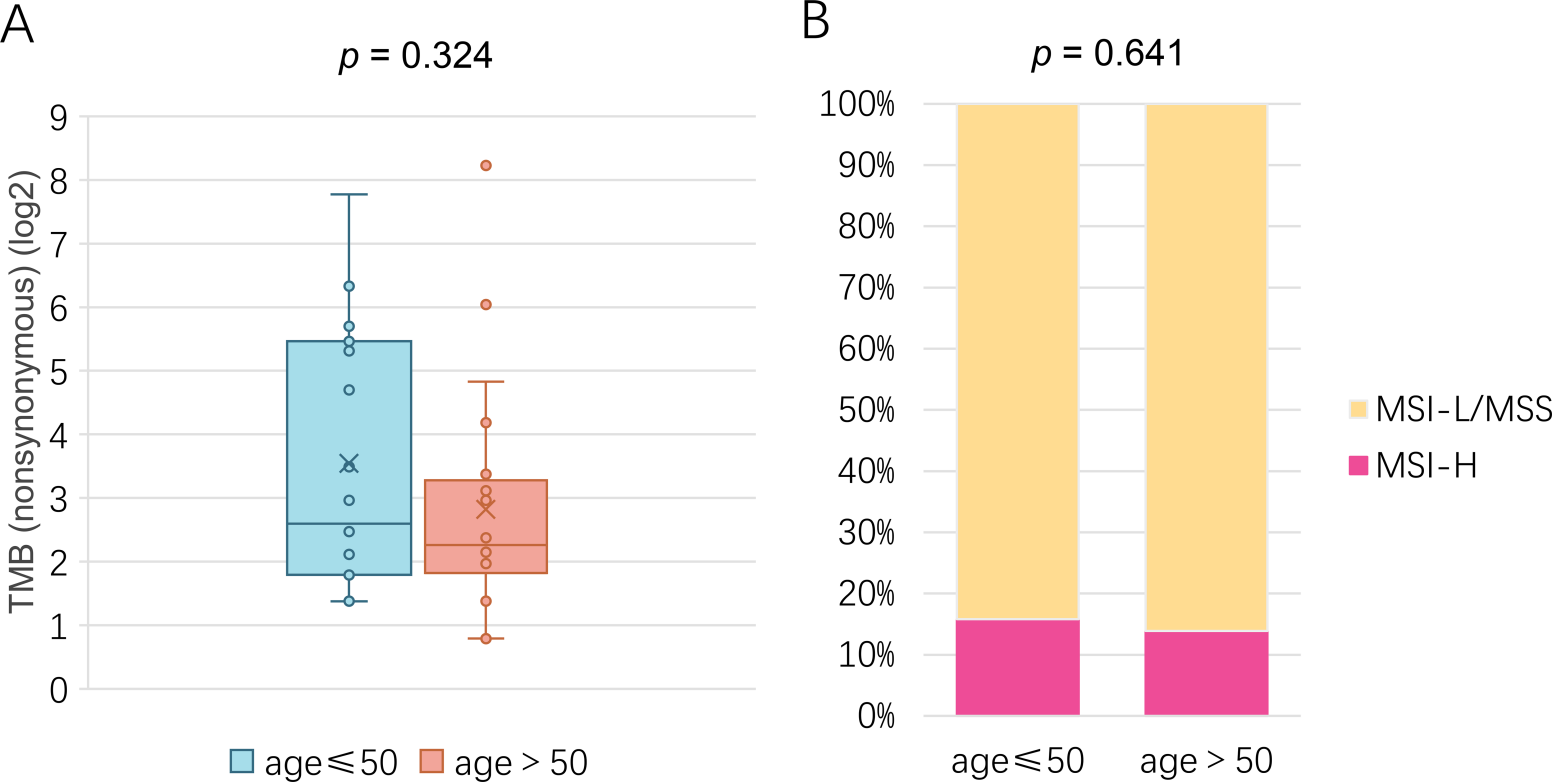
Association between and age in in MSK-MET. **(A)** Correlation analysis between TMB and age in asian patients with EC. **(B)** Comparison of MSI scroe between young and old asian patients with EC.

Analysis of Tumor Mutation Burden (TMB) revealed no statistically significant difference between the young and old patient cohorts (*p* = 0.324, **Figure 4A**). Descriptive statistics further illustrated this similarity. The mean TMB was nearly identical at 30.00 mutations/Mb (SD = ± 49.65) in the young group compared to 31.86 mutations/Mb (SD = ± 90.27) in the old group. Notably, the median TMB was precisely the same at 6.05 mutations/Mb for both cohorts. However, the elderly group exhibited a substantially wider range (1.73 - 549.95 mutations/Mb) and a larger standard deviation, suggesting greater heterogeneity in TMB values compared to the younger group (range: 2.59 - 218.77 mutations/Mb).

We next compared the distribution of microsatellite instability (MSI) status between the two age groups. In the young group, 3 patients (15.8%) were classified as MSI-H, compared to 16 (84.2%) as MSI-L/MSS. Similarly, the old group comprised 10 MSI-H patients (13.7%) and 63 MSI-L/MSS patients (84.9%), and 1 missing data (1.37%). Consistent with this distribution, statistical analysis confirmed that the difference in MSI scores between younger and elderly patients was not significant (*p* = 0.641, **Figure 4B**).

### 3.6 Survival outcomes

**Figure 5:**
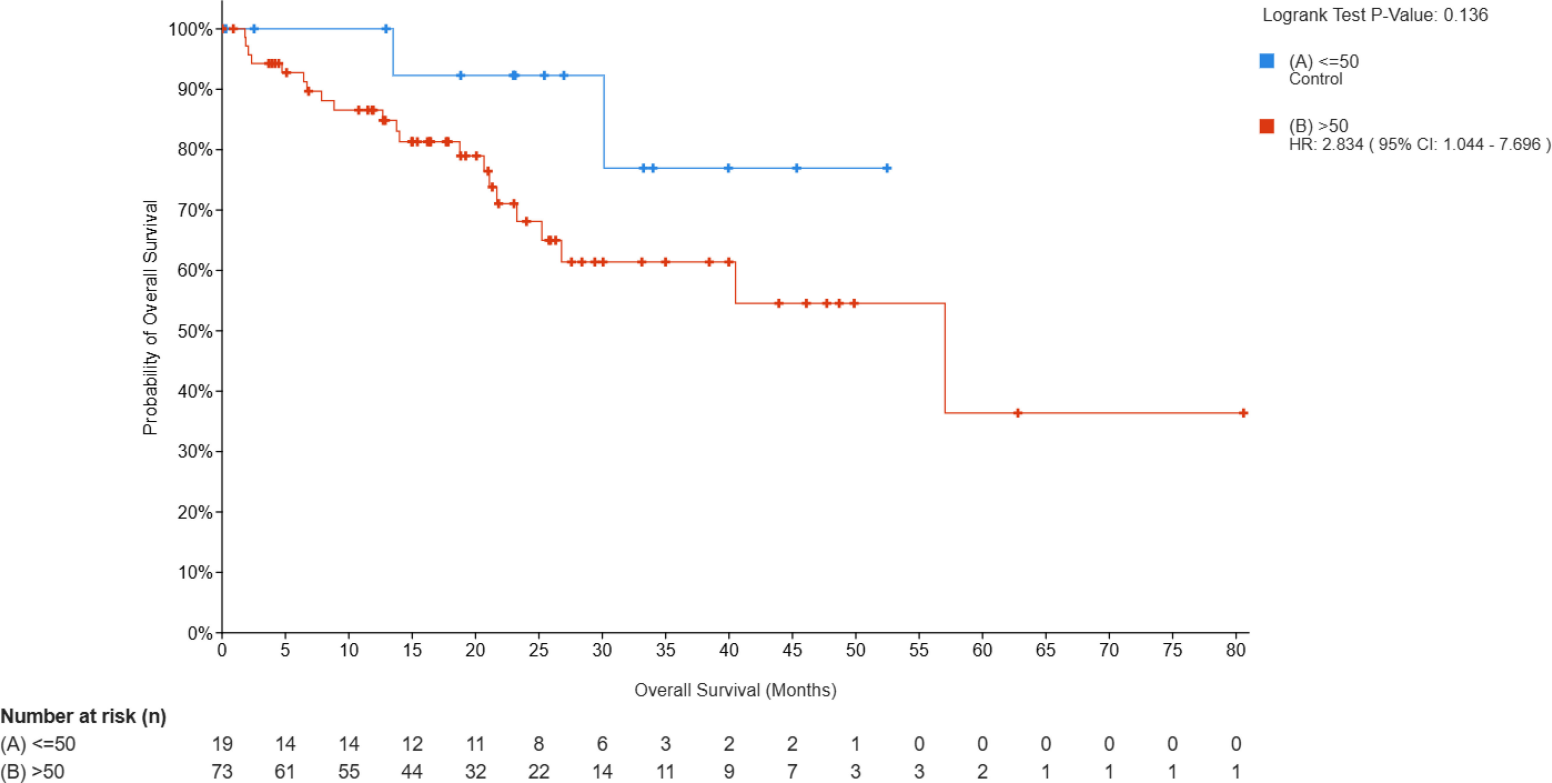
The overall survival of asian EC patients in MSK-MET.

Kaplan-Meier survival analysis revealed a pronounced age-dependent disparity in long-term clinical outcomes among endometrial carcinoma (EC) patients. Younger patients demonstrated significantly superior overall survival compared to elderly counterparts, with5-year survival rates of 76.92% versus 36.38%, respectively HR: 0.353(95% CI:0.130-0.985, *p* = 0.136) (**Figure 5**). The substantial survival difference observed between the young and elderly EC patients may reflect a true clinical disparity, though the lack of statistical significance, likely due to the limited sample size, warrants further investigation with larger cohorts.

### 3.7 Genomic profile of EC in EC-3rd-SYSU cohort

Given this context, we sought to determine if these observations could be generalized to a specific patient population. Consequently, we conducted an identical analysis within our internal cohort, the EC-3rd-SYSU dataset (n=18), which comprises genomic data from Chinese endometrial carcinoma patients.

**Figure 6:**
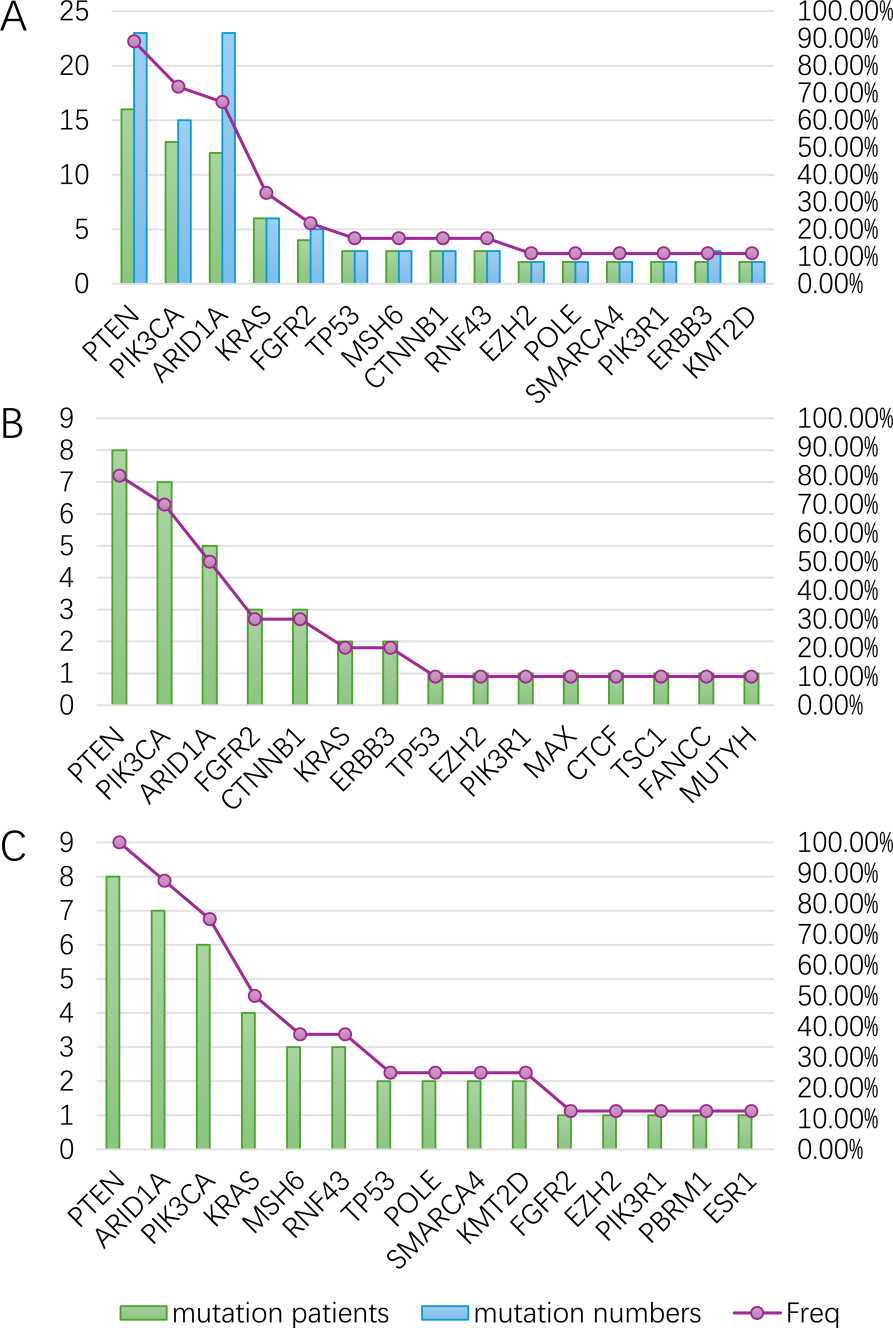
**(A)** Landscape of genomic alterations among EC-3rd-SYSU cohort. The comparative analysis of frequencies of gene mutations between young **(B)** and old **(C)** groups with EC.

The most frequently mutated 5 genes were PTEN (88.89%; total number of mutations, n=23; number of samples with one or more mutations, n=16), PIK3CA (72.22%; n=15; n=13), ARID1A (66.67%; n=23; n=12), KRAS (33.33%; n=6; n=6), and FGFR2 (22.22%; n=5; n=4). These were followed by genes ranked 6-15 — including TP53 (16.67%), MSH6 (16.67%), CTNNB1 (16.67%), RNF43 (16.67%) and others — with mutation frequencies ranging from 16.67% to 11.11%.” (**Figure 6A**).

A comparison of the genomic profiles from both cohorts confirms PTEN, PIK3CA and ARID1A as the most frequently mutated genes in endometrial carcinoma, consistent across populations. However, the EC-3rd-SYSU cohort showed notably higher PTEN mutation rates (88.89%) and greater prominence of KRAS and FGFR2 alterations, while TP53 and PIK3R1 mutations were more common in the MSK-MET cohort. These differences suggest potential population-specific molecular features that could inform targeted therapy strategies.

### 3.8 The clinical characteristics of EC-3rd-SYSU cohort

The patients were divided into two groups based on age: young group (age ≤50 years, 10 patients) and old group (age>50 years, 8 patients). In the EC-3rd-SYSU cohort, younger (n = 10) and older (n = 8) endometrial cancer patients differed significantly in mean age (43.80 ± 6.37 vs. 56.75 ± 2.12 years, p = 5.04E-05). However, no significant differences were detected in histological subtype (p = 0.358), pathological grading (p = 0.656), metastasis (p = 0.637), FIGO stage (p = 0.638), molecular subtype (p = 0.456), or MSI type (p = 0.410) (**Table 2**).

**Table 2:** Clinical characteristics of EC-3rd-SYSU cohort.

Characteristics	Young (n = 10)	Old (n = 8)	p value
Age (mean,SD)	43.80±6.37	56.75±2.12	5.04E-05
Histological subtype			0.358
Endometrioid Carcinoma	9	7	
Clear cell carcinoma	1	0	
Undifferentiated carcinoma	0	1	
Pathological Grading			0.656
grade1	7	4	
grade2	0	1	
grade3	2	2	
NA	1	1	
Metastasis			0.637
yes	4	5	
no	6	3	
FIGO stage			0.638
I	4	2	
II	2	1	
III	4	5	
IV	0	0	
Molecular subtype			0.456
POLE	0	1	
MMRd	2	3	
NSMP	7	3	
p53	1	1	
MSI Type			0.410
Stable	8	5	
Instable	2	3	
TMB (mean,SD)	17.02±21.55	20.05±15.18	0.741

### 3.9 Differences in mutation spectrums between young and old EC in EC-3rd-SYSU cohort

Among young patients, the most frequently mutated 5 genes were PTEN (80.00%, n=8), PIK3CA (70.00%, n=8), ARID1A (50.00%, n=5), FGFR2 (30.00%, n=3) and CTNNB1 (30.00%, n=3), (**Figure 6B**). In elderly patients, mutations in genes such as PTEN (100.00%, number of samples with one or more mutations, n=8), ARID1A (87.50%, n=7), PIK3CA (75.00%, n=6), KRAS (50.00%, n=4), and MSH6 (37.5%, n=3) (**Figure 6C**; **Supplementary Table S3**) were more prevalent.

**Figure 7:**
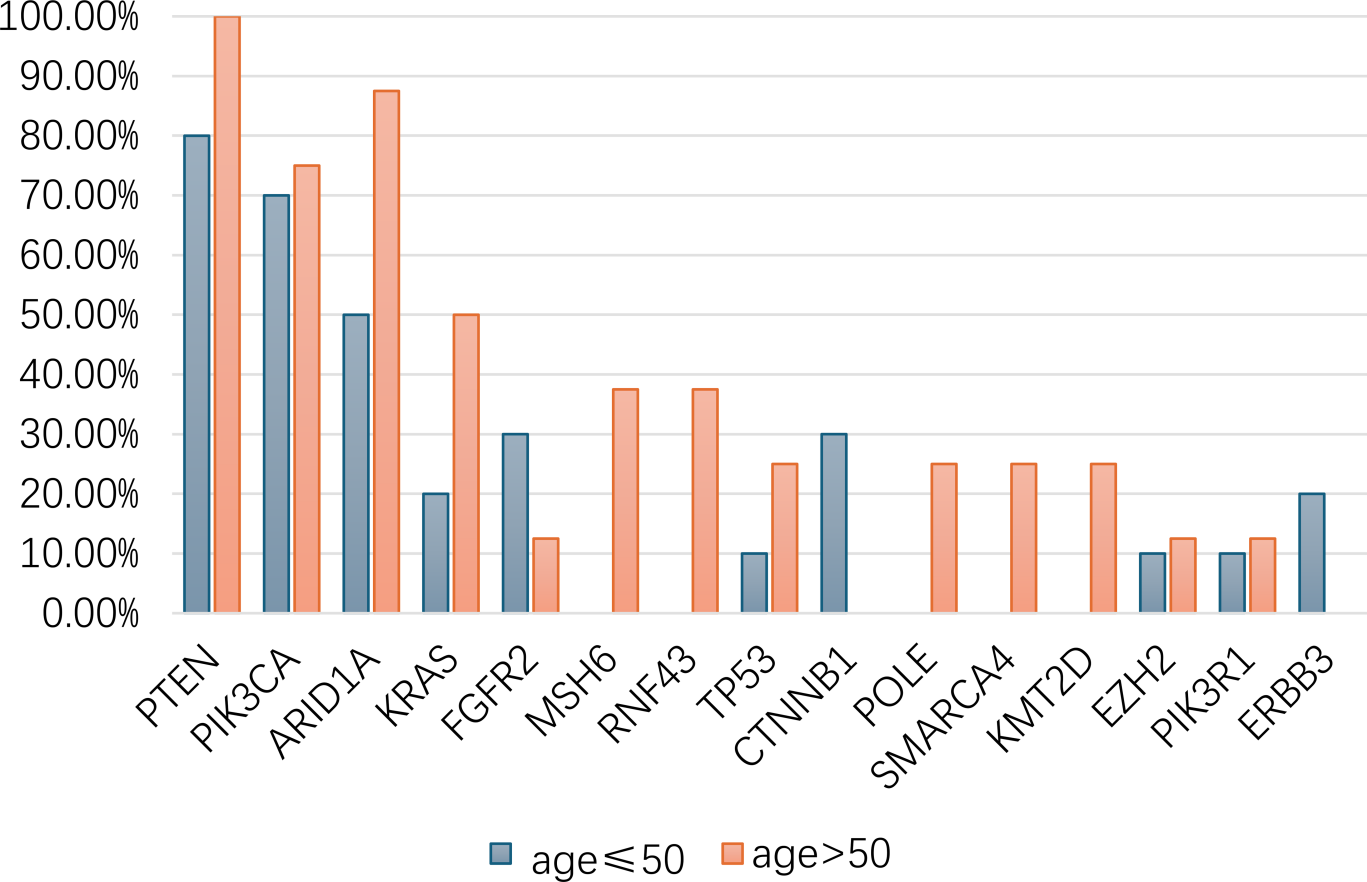
Genomic profiling between young and old patients in EC-3rd-SYSU cohort. No significant difference was found in this cohort.

Statistical analysis revealed that the frequencies of CTNNB1, FGFE2 and ERBB3 mutations in the young group were higher compared with that in the old group (30.00% vs 0%, 30.00% vs 12.50% and 20.00% vs 0%, respectively), while ARID1A, KRAS, MSH6, RNF43 alterations were associated with old age (87.50% vs 50.00%, 50.00% vs 20.00%, 37.5% vs 0% and 37.5% vs 0%), (**Figure 7**). However, the small sample size resulted in low statistical power, limiting our ability to detect significant differences.

Comparing the age-related mutation spectra across two cohorts reveals consistent biological patterns. CTNNB1 mutations consistently show age-specific distributions, while PTEN maintains high frequency in younger patients across both populations. Notably, TP53 enrichment in older MSK-MET patients finds parallel in our cohort with ARID1A, KRAS and MSH6 showing similar age-associated trends. These conserved patterns suggest fundamental age-related molecular mechanisms in endometrial carcinogenesis that transcend ethnic boundaries.

### 3.10 Pathways and molecular mechanisms in EC-3rd-SYSU cohort

To investigate the biological implications of age-related genomic differences in EC, we analyzed the 15 most frequently mutated genes (**Figures 6B, C**), followed by KEGG pathway enrichment analysis for each group.

**Figure 8:**
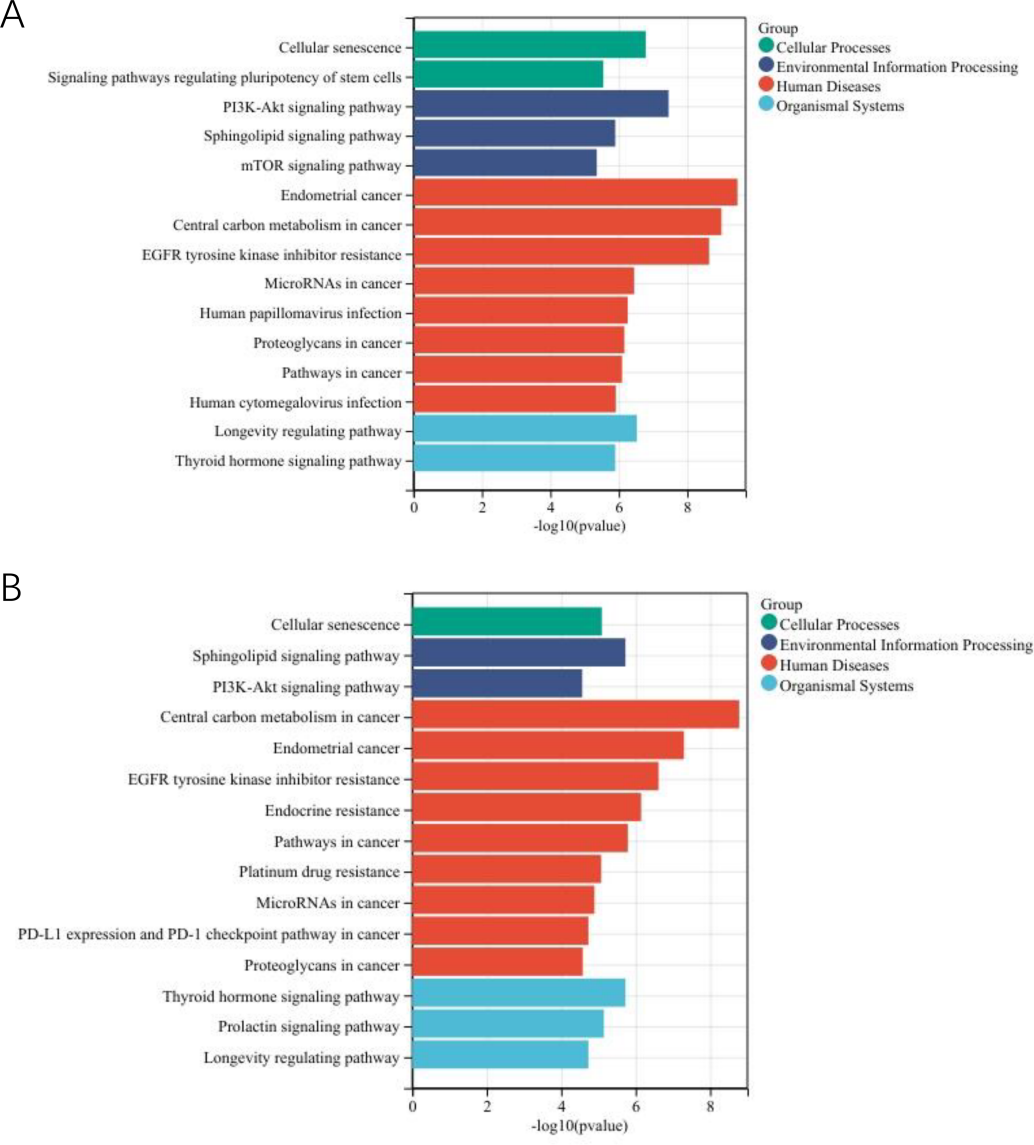
The most enriched KEGG pathways associated with actionable mutations in EC- 3rd-SYSU cohort. **(A)** The enriched pathways in the young patients with EC. **(B)** The enriched pathways in the old patients with EC.

Enrichment analysis revealed significant terms across multiple categories in young group: for cellular processes, cellular senescence and signaling pathways regulating pluripotency of stem cells; for environmental information processing, PI3K-Akt signaling pathway, sphingolipid signaling pathway and mTOR signaling pathway; for human diseases, endometrial cancer, central carbon metabolism in cancer, microRNAs in cancer, EGFR tyrosine kinase inhibitor resistance, human papillomavirus infection, proteoglycans in cancer, pathways in cancer and human cytomegalovirus infection; and for organismal systems, longevity regulating pathway and thyroid hormone signaling pathway (**Figure 8A**).

In old group, cellular processes were enriched for cellular senescence. Environmental information processing was enriched for PI3K-Akt signaling pathway and sphingolipid signaling pathway; Human diseases featured central carbon metabolism in cancer, EGFR tyrosine kinase inhibitor resistance, endometrial cancer, endocrine resistance, pathways in cancer, platinum drug resistance, microRNAs in cancer, PD-L1 expression and PD-1 checkpoint pathway in cancer and human T-cell leukemia virus 1 infection. These processes were anchored in organismal systems including thyroid hormone signaling pathway, prolactin signaling pathway and longevity regulating pathway (**Figure 8B**).

Pathway enrich analysis of the two cohorts revealed conserved age-related pathway distinctions. Younger patients consistently exhibited enrichment in pathways governing developmental plasticity (e.g., stem cell pluripotency), whereas older patients showed preferential involvement of therapy resistance pathways (e.g., endocrine resistance, platinum drug resistance). A key inter-cohort difference was the specific activation of PI3K-Akt/mTOR signaling in younger patients of the EC-3rd-SYSU cohort, suggesting potential population-specific oncogenic mechanisms in this age group.

### 3.11 Age-stratified genomic instability correlates in EC-3rd-SYSU cohort

**Figure 9:**
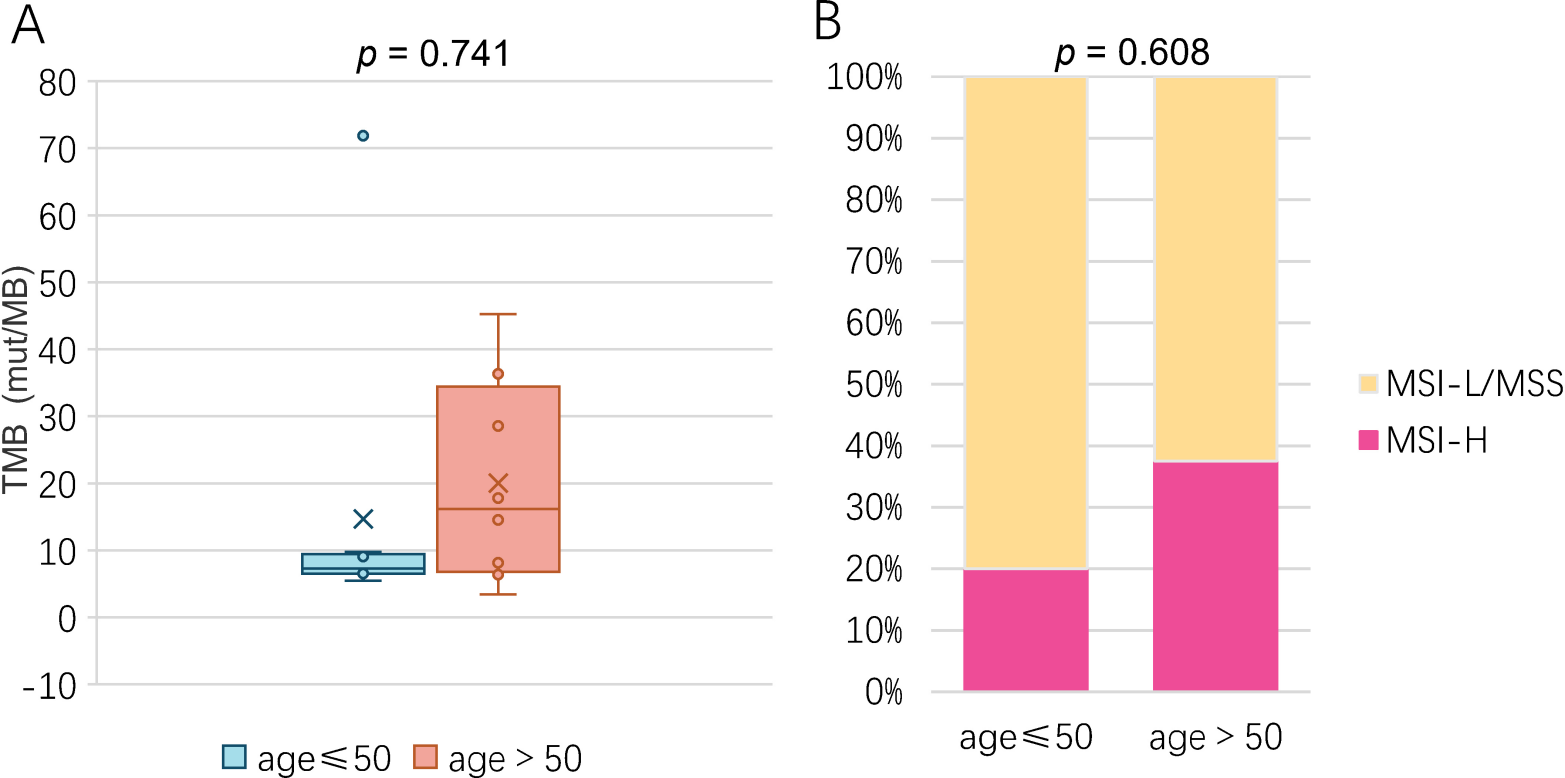
Association between TMB and age in EC-3rd-SYSU cohort. **(A)** Correlation analysis between TMB and age in asia patients with EC. **(B)** Comparison of MSI scroe between young and old asian patients with EC.

Analysis of Tumor Mutation Burden (TMB) revealed no statistically significant difference between the young and old patient cohorts (*p* = 0.741, **Figure 9A**). Descriptive statistics further illustrated this similarity. The mean TMB was nearly identical at 17.02 ± 21.55 mutations/Mb in the young group compared to 20.05 ± 15.18 mutations/Mb in the old group.

We next compared the distribution of microsatellite instability (MSI) status between the two age groups. In the young group, 2 patients (20.0%) were classified as MSI-H, compared to 8 (80.0%) as MSI-L/MSS. Similarly, the old group comprised 3 MSI-H patients (37.5%) and 5 MSI-L/MSS patients (62.5%). Consistent with this distribution, statistical analysis confirmed that the difference in MSI scores between younger and elderly patients was not significant (*p* = 0.608, **Figure 9B**).

## 4 Discussion

Endometrial carcinoma is a heterogeneous disease with distinct molecular and clinical characteristics based on various factors, including age (14). Previous studies have shown that while EC is typically diagnosed in menopausal women, an increasing number of young women are presenting with this malignancy (15, 16). The findings of our study underscore that young and elderly patients with EC exhibit significantly different genomic profiles, reflecting how age influences tumor biology. However, due to the small sample size of the EC-3rd-SYSU cohort (n=18), statistical power is limited and the findings require cautious interpretation. These results should therefore be considered exploratory and validated in larger, independent Asian cohorts.

Distinct age-associated mutational patterns were observed within both cohorts. In the MSK-MET cohort, *CTNNB1* and *PTEN* mutations were markedly enriched in younger patients, while *TP53* alterations predominated in older individuals. A similar trend was noted in the EC-3rd-SYSU cohort, where younger patients showed higher frequencies of *CTNNB1* and *FGFR2* mutations, contrasting with the enrichment of *ARID1A*, *KRAS*, and *MSH6* mutations in older patients. These results suggest divergent tumorigenic trajectories shaped by age-dependent molecular mechanisms. While *TP53* loss-of-function compromises genomic surveillance mechanisms, constitutive PI3K-Akt activation promotes metabolic reprogramming and therapy resistance, collectively fostering aggressive tumor phenotypes (17, 18). In older individuals, age-related genomic instability—driven by declining DNA repair and a pro-inflammatory microenvironment with cellular senescence—likely fosters the accumulation of TP53/ARID1A mutations and selects for clones adapted to this stress (19). In contrast, tumorigenesis in younger patients may be more directly driven by strong activation of specific pathways in a setting of more intact genomic maintenance (20). These findings rationalize the clinical failure of conventional therapies in elderly EC and advocate for combinatorial regimens targeting both pathways. Notably, PARP inhibitors in *TP53*-mutated contexts paired with PI3Kβ-sparing inhibitors may achieve synthetic lethality while minimizing toxicity in frail elderly patients (21).

The observed molecular distinctions between younger and older patients may be partially influenced by underlying germline susceptibility, which constitutes an important non-age-related confounder. Notably, pathogenic germline variants in genes associated with hereditary cancer syndromes, such as Lynch syndrome (MLH1, MSH2, MSH6, PMS2), are established risk factors for EC and are more frequently identified in women diagnosed at a younger age (1). In our analysis of the MSK-MET cohort, we observed a non-significant trend towards a higher prevalence of pathogenic/likely pathogenic germline variants in Lynch syndrome-associated genes among patients diagnosed before age 50 compared to those diagnosed after age 50 (MLH1, 10.53% vs. 6.85%; p=0.63; MSH2, 10.53% vs. 5.48%; p=0.60; MSH6, 15.79% vs. 8.22%; p=0.39; PMS2, 10.53% vs. 4.11%; p=0.27) (**Supplementary Table S2**). This aligns with prior reports suggesting that a proportion of early-onset EC cases arise in the context of inherited susceptibility (10). However, a comprehensive germline assessment was not feasible in our validation cohort (EC-3rd-SYSU), and that incomplete ascertainment of germline status remains a limitation of our study.

While both cohorts displayed a conserved driver landscape, notable population-specific distinctions were evident. The Chinese EC-3rd-SYSU cohort exhibited a higher overall mutation burden and distinct prominence of KRAS and FGFR2 mutations, whereas TP53 and PIK3R1 alterations were more prevalent in the MSK-MET dataset. These findings suggest potential population or ethnicity-associated genomic differences that may influence tumor biology, as previously proposed in large-scale molecular epidemiologic analyses (22). The enrichment of KRAS and FGFR2 in the Chinese cohort could indicate a stronger contribution of receptor tyrosine kinase–RAS signaling to tumorigenesis, providing potential therapeutic opportunities with FGFR or MEK inhibitors like in other cancers (23). Concurrently, distinct environmental exposures, such as severe air pollution, have been shown to directly reshape the mutational landscape, leading to an increased mutation burden in genes (24). Together, these results emphasize that while core oncogenic events are conserved, secondary mutation spectra may vary geographically or genetically, shaping distinct disease phenotypes and treatment responses across populations.

KEGG enrichment analyses in both cohorts revealed highly concordant age-related signaling architectures. Shared enrichment of oncogenic and endocrine pathways—such as endometrial cancer, central carbon metabolism in cancer, EGFR tyrosine kinase inhibitor resistance, and thyroid hormone signaling—demonstrates a conserved molecular backbone of EC. However, younger patients consistently showed activation of stemness-related and metabolic adaptability pathways (e.g., stem cell pluripotency, PI3K-Akt/mTOR signaling, insulin resistance), whereas older patients exhibited upregulation of cellular senescence, endocrine resistance, and longevity regulation pathways. These results align with recent integrative studies indicating that aging reprograms tumor metabolism and immune evasion through senescence-associated signaling networks (25). Notably, the specific activation of PI3K-Akt/mTOR signaling in younger Chinese patients may represent a cohort-specific oncogenic feature, reflecting both genetic predisposition and environmental modulation. Collectively, these findings illustrate a conserved yet contextually adaptive molecular framework of age-related tumor evolution in EC.

Biologically, TMB-H and MSI-H status reflect a hypermutated genome with increased neoantigen load, which enhances tumor immunogenicity and the likelihood of response to immune checkpoint inhibitors (ICIs) targeting the PD-1/PD-L1 axi (10). Clinically, MSI-H/dMMR is a validated predictive biomarker for ICIs in EC, underpinning the FDA approval of pembrolizumab for advanced MSI-H solid tumors based on the KEYNOTE-158 trial and establishing lenvatinib plus pembrolizumab as a standard therapy in KEYNOTE-775 (26). Furthermore, TMB-H serves as a complementary biomarker, with evidence suggesting that EC patients with TMB-H tumors, including a subset of MSS cases, may derive greater benefit from immunotherapy (27). Our evaluation of genomic instability, measured by tumor TMB and MSI status, revealed no statistically significant differences between young and old patients in either cohort, suggesting that neither overall mutational load nor mismatch repair deficiency primarily drives the age-related survival disparity. Previous studies have shown inconsistent associations between age and TMB, with some reporting increases in older individuals but a non-linear pattern across age groups (28, 29). Similarly, MSI-H frequency appears in old patients in solid tumors, suggesting that MSI represents a key carcinogenic pathway in age-related cancers (30). However, given the relatively limited sample size in our cohorts, the absence of significant differences should be interpreted with caution, as subtle age-related genomic effects might not have been detected with the current statistical power.

## 5 Conclusion

Our study reveals a conserved genomic landscape in endometrial carcinoma across Asian (partially Chinese) populations, dominated by PTEN, PIK3CA, and ARID1A mutations. Despite shared core pathways, distinct age- and population-specific features were identified. Younger patients exhibited enrichment in CTNNB1 and PTEN mutations and activation of PI3K-Akt/mTOR and stemness-related signaling, while older patients showed higher TP53 and ARID1A mutation frequencies alongside senescence and endocrine resistance pathways. The prominence of KRAS and FGFR2 mutations in the Chinese cohort further suggests ethnic variation in oncogenic drivers. Collectively, these findings underscore the need for age- and population-tailored therapeutic strategies in precision management of endometrial carcinoma.

## Data Availability

The MSK-MET datasets presented in this study can be found in online repositories (https://www.cbioportal.org/). The EC-3rd-SYSU data are available from the corresponding author on reasonable request.
