# Supplementary material for: Genomic and immunological differences in endometrial cancer: a comparative study between young and old Asian patients
Source: Front Immunol. 2026 Feb 10;17:1737794. doi: 10.3389/fimmu.2026.1737794 (PMC12929549; doi:10.3389/fimmu.2026.1737794)
Supplement: Supplementary file 1 [file Table1.docx]

Table S1. The list of 90 genes in the customized gene panel

| ABRAXAS1 | AKT1 | AKT2 | AKT3 | ALK | ATM | ATR | AR | ARAF | ARID1A |
| --- | --- | --- | --- | --- | --- | --- | --- | --- | --- |
| B2M | BAP1 | BARD1 | BRAF | BRCA1 | BRCA2 | BRIP1 | C11ORF30 (EMSY) | CDK4 | CDK6 |
| CDK12 | CDKN2A | CHEK1 | CHEK2 | CTNNB1 | EGFR | EPCAM | ERBB2 | ESR1 | ESR2 |
| FANCA | FANCC | FANCD2 | FANCI | FANCL | FGF19 | FGFR1 | FGFR2 | FGFR3 | FGFR4 |
| FLT1 | FLT4 | HOXB13 | HRAS | IDH1 | IDH2 | JAK1 | JAK2 | KDR | KIT |
| KRAS | MAP2K1 | MDM2 | MDM4 | MET | MLH1 | MRE11 | MSH2 | MSH6 | MTOR |
| NBN | NF1 | NF2 | NRAS | NTRK1 | NTRK2 | NTRK3 | PALB2 | PBRM1 | PDGFRA |
| PGR | PIK3CA | PMS2 | POLD1 | POLE | PPP2R2A | PTEN | RAD50 | RAD51B | RAD51C |
| RAD51D | RAD54B | RAD54L | RB1 | RET | ROS1 | STK11 | TP53 | TSC1 | TSC |

Table S2. Gene mutation data from the MSK-MET cohort

| Gene | Young group  count(ratio) | Old group  count(ratio) | Log2 Ratio  (young vs old) | p-Value |
| --- | --- | --- | --- | --- |
| CTNNB1 | 13 (68.42%) | 9 (12.33%) | 2.47 | 3.03E-06 |
| TP53 | 3 (15.79%) | 40 (54.79%) | -1.8 | 3.73E-03 |
| PTEN | 16 (84.21%) | 37 (50.68%) | 0.73 | 9.35E-03 |
| SPOP | 5 (26.32%) | 5 (6.85%) | 1.94 | 0.0288 |
| NTRK1 | 4 (21.05%) | 4 (5.48%) | 1.94 | 0.0539 |
| EZH2 | 3 (15.79%) | 2 (2.74%) | 2.53 | 0.0578 |
| NOTCH1 | 0 (0.00%) | 12 (16.44%) | <-10 | 0.0657 |
| FAT1 | 6 (31.58%) | 9 (12.33%) | 1.36 | 0.0749 |
| MED12 | 6 (31.58%) | 9 (12.33%) | 1.36 | 0.0749 |
| NSD1 | 4 (21.05%) | 5 (6.85%) | 1.62 | 0.0837 |
| TNFAIP3 | 3 (15.79%) | 3 (4.11%) | 1.94 | 0.1 |
| HGF | 2 (10.53%) | 1 (1.37%) | 2.94 | 0.107 |
| PAX5 | 2 (10.53%) | 1 (1.37%) | 2.94 | 0.107 |
| RYBP | 2 (10.53%) | 1 (1.37%) | 2.94 | 0.107 |
| ANKRD11 | 5 (27.78%) | 8 (11.43%) | 1.28 | 0.129 |
| EPHB1 | 3 (15.79%) | 4 (5.48%) | 1.53 | 0.152 |
| GRIN2A | 3 (15.79%) | 4 (5.48%) | 1.53 | 0.152 |
| NSD3 | 3 (17.65%) | 4 (6.45%) | 1.45 | 0.166 |
| FGFR2 | 1 (5.26%) | 15 (20.55%) | -1.96 | 0.177 |
| STAT5B | 2 (11.11%) | 2 (2.86%) | 1.96 | 0.184 |
| BMPR1A | 2 (10.53%) | 2 (2.74%) | 1.94 | 0.188 |
| CHEK2 | 2 (10.53%) | 2 (2.74%) | 1.94 | 0.188 |
| DIS3 | 2 (10.53%) | 2 (2.74%) | 1.94 | 0.188 |
| HNF1A | 2 (10.53%) | 2 (2.74%) | 1.94 | 0.188 |
| MITF | 2 (10.53%) | 2 (2.74%) | 1.94 | 0.188 |
| CCNE1 | 0 (0.00%) | 9 (12.33%) | <-10 | 0.195 |
| ABL1 | 0 (0.00%) | 8 (10.96%) | <-10 | 0.198 |
| ERCC3 | 0 (0.00%) | 8 (10.96%) | <-10 | 0.198 |
| ERF | 2 (11.76%) | 2 (3.23%) | 1.87 | 0.201 |
| PLCG2 | 4 (22.22%) | 6 (8.57%) | 1.37 | 0.204 |
| NEGR1 | 1 (5.56%) | 0 (0.00%) | >10 | 0.205 |
| CREBBP | 4 (21.05%) | 6 (8.22%) | 1.36 | 0.206 |
| TET1 | 4 (21.05%) | 6 (8.22%) | 1.36 | 0.206 |
| COP1 | 1 (5.26%) | 0 (0.00%) | >10 | 0.207 |
| CTLA4 | 1 (5.26%) | 0 (0.00%) | >10 | 0.207 |
| NRAS | 1 (5.26%) | 0 (0.00%) | >10 | 0.207 |
| PIM1 | 1 (5.26%) | 0 (0.00%) | >10 | 0.207 |
| ELF3 | 1 (5.88%) | 0 (0.00%) | >10 | 0.215 |
| SESN2 | 1 (5.88%) | 0 (0.00%) | >10 | 0.215 |
| SETD2 | 4 (21.05%) | 7 (9.59%) | 1.13 | 0.229 |
| ACVR1 | 2 (11.11%) | 3 (4.29%) | 1.37 | 0.27 |
| AXIN2 | 2 (10.53%) | 3 (4.11%) | 1.36 | 0.274 |
| CDH1 | 2 (10.53%) | 3 (4.11%) | 1.36 | 0.274 |
| CSF1R | 2 (10.53%) | 3 (4.11%) | 1.36 | 0.274 |
| ESR1 | 2 (10.53%) | 3 (4.11%) | 1.36 | 0.274 |
| NTRK3 | 2 (10.53%) | 3 (4.11%) | 1.36 | 0.274 |
| PMS2 | 2 (10.53%) | 3 (4.11%) | 1.36 | 0.274 |
| RAF1 | 2 (10.53%) | 3 (4.11%) | 1.36 | 0.274 |
| ARID5B | 5 (26.32%) | 10 (13.70%) | 0.94 | 0.292 |
| MAP3K1 | 5 (26.32%) | 10 (13.70%) | 0.94 | 0.292 |
| LYN | 0 (0.00%) | 6 (9.68%) | <-10 | 0.331 |
| IKZF1 | 0 (0.00%) | 7 (9.59%) | <-10 | 0.338 |
| KEAP1 | 0 (0.00%) | 7 (9.59%) | <-10 | 0.338 |
| PTPRS | 0 (0.00%) | 7 (9.59%) | <-10 | 0.338 |
| GATA2 | 0 (0.00%) | 6 (8.22%) | <-10 | 0.339 |
| IRS1 | 0 (0.00%) | 6 (8.22%) | <-10 | 0.339 |
| PARP1 | 0 (0.00%) | 6 (8.22%) | <-10 | 0.339 |
| SDHA | 0 (0.00%) | 6 (8.22%) | <-10 | 0.339 |
| STK11 | 0 (0.00%) | 6 (8.22%) | <-10 | 0.339 |
| POLD1 | 0 (0.00%) | 6 (8.57%) | <-10 | 0.339 |
| TCF3 | 0 (0.00%) | 6 (8.57%) | <-10 | 0.339 |
| KDM5A | 3 (15.79%) | 5 (6.85%) | 1.2 | 0.355 |
| LATS1 | 3 (15.79%) | 5 (6.85%) | 1.2 | 0.355 |
| MAPK3 | 1 (5.56%) | 1 (1.43%) | 1.96 | 0.369 |
| CHEK1 | 1 (5.26%) | 1 (1.37%) | 1.94 | 0.372 |
| FGF19 | 1 (5.26%) | 1 (1.37%) | 1.94 | 0.372 |
| H3-5 | 1 (5.26%) | 1 (1.37%) | 1.94 | 0.372 |
| NKX3-1 | 1 (5.26%) | 1 (1.37%) | 1.94 | 0.372 |
| RAC1 | 1 (5.26%) | 1 (1.37%) | 1.94 | 0.372 |
| SDHD | 1 (5.26%) | 1 (1.37%) | 1.94 | 0.372 |
| SH2D1A | 1 (5.26%) | 1 (1.37%) | 1.94 | 0.372 |
| SUZ12 | 1 (5.26%) | 1 (1.37%) | 1.94 | 0.372 |
| TGFBR2 | 1 (5.26%) | 1 (1.37%) | 1.94 | 0.372 |
| YAP1 | 1 (5.26%) | 1 (1.37%) | 1.94 | 0.372 |
| DICER1 | 3 (15.79%) | 6 (8.22%) | 0.94 | 0.385 |
| EP300 | 3 (15.79%) | 6 (8.22%) | 0.94 | 0.385 |
| ERBB3 | 3 (15.79%) | 6 (8.22%) | 0.94 | 0.385 |
| FLT4 | 3 (15.79%) | 6 (8.22%) | 0.94 | 0.385 |
| KDR | 3 (15.79%) | 6 (8.22%) | 0.94 | 0.385 |
| MSH6 | 3 (15.79%) | 6 (8.22%) | 0.94 | 0.385 |
| STAG2 | 3 (15.79%) | 6 (8.22%) | 0.94 | 0.385 |
| EZH1 | 1 (5.88%) | 1 (1.61%) | 1.87 | 0.386 |
| MAPKAP1 | 1 (5.88%) | 1 (1.61%) | 1.87 | 0.386 |
| PDCD1LG2 | 1 (5.88%) | 1 (1.61%) | 1.87 | 0.386 |
| PPARG | 1 (5.88%) | 1 (1.61%) | 1.87 | 0.386 |
| PPP4R2 | 1 (5.88%) | 1 (1.61%) | 1.87 | 0.386 |
| PTP4A1 | 1 (5.88%) | 1 (1.61%) | 1.87 | 0.386 |
| RXRA | 1 (5.88%) | 1 (1.61%) | 1.87 | 0.386 |
| STK19 | 1 (5.88%) | 1 (1.61%) | 1.87 | 0.386 |
| ATRX | 3 (15.79%) | 7 (9.59%) | 0.72 | 0.425 |
| BLM | 3 (15.79%) | 7 (9.59%) | 0.72 | 0.425 |
| PTPRT | 3 (15.79%) | 7 (9.59%) | 0.72 | 0.425 |
| RNF43 | 3 (15.79%) | 7 (9.59%) | 0.72 | 0.425 |
| PIK3CA | 12 (63.16%) | 38 (52.05%) | 0.28 | 0.446 |
| PPP2R1A | 1 (5.26%) | 10 (13.70%) | -1.38 | 0.449 |
| KMT2D | 4 (21.05%) | 10 (13.70%) | 0.62 | 0.477 |
| SPEN | 4 (21.05%) | 10 (13.70%) | 0.62 | 0.477 |
| EPHA7 | 1 (5.56%) | 2 (2.86%) | 0.96 | 0.501 |
| ERRFI1 | 1 (5.56%) | 2 (2.86%) | 0.96 | 0.501 |
| RAB35 | 1 (5.56%) | 2 (2.86%) | 0.96 | 0.501 |
| VEGFA | 1 (5.56%) | 2 (2.86%) | 0.96 | 0.501 |
| ATM | 4 (21.05%) | 11 (15.07%) | 0.48 | 0.503 |
| INPPL1 | 2 (11.76%) | 13 (20.97%) | -0.83 | 0.503 |
| ALOX12B | 1 (5.26%) | 2 (2.74%) | 0.94 | 0.505 |
| AURKA | 1 (5.26%) | 2 (2.74%) | 0.94 | 0.505 |
| B2M | 1 (5.26%) | 2 (2.74%) | 0.94 | 0.505 |
| BTK | 1 (5.26%) | 2 (2.74%) | 0.94 | 0.505 |
| CDK6 | 1 (5.26%) | 2 (2.74%) | 0.94 | 0.505 |
| ETV6 | 1 (5.26%) | 2 (2.74%) | 0.94 | 0.505 |
| FANCC | 1 (5.26%) | 2 (2.74%) | 0.94 | 0.505 |
| GSK3B | 1 (5.26%) | 2 (2.74%) | 0.94 | 0.505 |
| INPP4B | 1 (5.26%) | 2 (2.74%) | 0.94 | 0.505 |
| JUN | 1 (5.26%) | 2 (2.74%) | 0.94 | 0.505 |
| MRE11 | 1 (5.26%) | 2 (2.74%) | 0.94 | 0.505 |
| MYCN | 1 (5.26%) | 2 (2.74%) | 0.94 | 0.505 |
| PALB2 | 1 (5.26%) | 2 (2.74%) | 0.94 | 0.505 |
| RPS6KA4 | 1 (5.26%) | 2 (2.74%) | 0.94 | 0.505 |
| SF3B1 | 1 (5.26%) | 2 (2.74%) | 0.94 | 0.505 |
| SOX2 | 1 (5.26%) | 2 (2.74%) | 0.94 | 0.505 |
| KMT2C | 2 (10.53%) | 14 (19.18%) | -0.87 | 0.509 |
| DUSP4 | 1 (5.88%) | 2 (3.23%) | 0.87 | 0.522 |
| NUF2 | 1 (5.88%) | 2 (3.23%) | 0.87 | 0.522 |
| PRKCI | 1 (5.88%) | 2 (3.23%) | 0.87 | 0.522 |
| RRAS2 | 1 (5.88%) | 2 (3.23%) | 0.87 | 0.522 |
| SESN3 | 1 (5.88%) | 2 (3.23%) | 0.87 | 0.522 |
| SHOC2 | 1 (5.88%) | 2 (3.23%) | 0.87 | 0.522 |
| CTCF | 5 (26.32%) | 14 (19.18%) | 0.46 | 0.53 |
| KRAS | 3 (15.79%) | 18 (24.66%) | -0.64 | 0.547 |
| CYLD | 0 (0.00%) | 4 (6.45%) | <-10 | 0.572 |
| BAP1 | 0 (0.00%) | 4 (5.48%) | <-10 | 0.577 |
| CD79B | 0 (0.00%) | 4 (5.48%) | <-10 | 0.577 |
| CDKN2A | 0 (0.00%) | 4 (5.48%) | <-10 | 0.577 |
| CUL3 | 0 (0.00%) | 4 (5.48%) | <-10 | 0.577 |
| E2F3 | 0 (0.00%) | 4 (5.48%) | <-10 | 0.577 |
| EIF1AX | 0 (0.00%) | 4 (5.48%) | <-10 | 0.577 |
| FLCN | 0 (0.00%) | 4 (5.48%) | <-10 | 0.577 |
| FOXP1 | 0 (0.00%) | 4 (5.48%) | <-10 | 0.577 |
| GNAQ | 0 (0.00%) | 4 (5.48%) | <-10 | 0.577 |
| GNAS | 0 (0.00%) | 4 (5.48%) | <-10 | 0.577 |
| IRF4 | 0 (0.00%) | 4 (5.48%) | <-10 | 0.577 |
| MPL | 0 (0.00%) | 4 (5.48%) | <-10 | 0.577 |
| MUTYH | 0 (0.00%) | 4 (5.48%) | <-10 | 0.577 |
| PAK5 | 0 (0.00%) | 4 (5.48%) | <-10 | 0.577 |
| PRKN | 0 (0.00%) | 4 (5.48%) | <-10 | 0.577 |
| RBM10 | 0 (0.00%) | 4 (5.48%) | <-10 | 0.577 |
| SMAD2 | 0 (0.00%) | 4 (5.48%) | <-10 | 0.577 |
| TGFBR1 | 0 (0.00%) | 4 (5.48%) | <-10 | 0.577 |
| TSHR | 0 (0.00%) | 4 (5.48%) | <-10 | 0.577 |
| DNAJB1 | 0 (0.00%) | 4 (5.71%) | <-10 | 0.577 |
| CEBPA | 0 (0.00%) | 5 (7.14%) | <-10 | 0.579 |
| NSD2 | 0 (0.00%) | 5 (8.06%) | <-10 | 0.579 |
| UPF1 | 0 (0.00%) | 5 (8.06%) | <-10 | 0.579 |
| AKT3 | 0 (0.00%) | 5 (6.85%) | <-10 | 0.579 |
| AXIN1 | 0 (0.00%) | 5 (6.85%) | <-10 | 0.579 |
| BCL6 | 0 (0.00%) | 5 (6.85%) | <-10 | 0.579 |
| BRD4 | 0 (0.00%) | 5 (6.85%) | <-10 | 0.579 |
| FANCA | 0 (0.00%) | 5 (6.85%) | <-10 | 0.579 |
| NBN | 0 (0.00%) | 5 (6.85%) | <-10 | 0.579 |
| SYK | 0 (0.00%) | 5 (6.85%) | <-10 | 0.579 |
| MSH2 | 2 (10.53%) | 4 (5.48%) | 0.94 | 0.6 |
| NOTCH2 | 2 (10.53%) | 4 (5.48%) | 0.94 | 0.6 |
| NTRK2 | 2 (10.53%) | 4 (5.48%) | 0.94 | 0.6 |
| PIK3R2 | 2 (10.53%) | 4 (5.48%) | 0.94 | 0.6 |
| PRDM1 | 2 (10.53%) | 4 (5.48%) | 0.94 | 0.6 |
| TP63 | 2 (10.53%) | 4 (5.48%) | 0.94 | 0.6 |
| XPO1 | 2 (10.53%) | 4 (5.48%) | 0.94 | 0.6 |
| TP53BP1 | 2 (11.76%) | 4 (6.45%) | 0.87 | 0.604 |
| ARID1A | 10 (52.63%) | 33 (45.21%) | 0.22 | 0.613 |
| SH2B3 | 2 (11.11%) | 5 (7.14%) | 0.64 | 0.628 |
| ATR | 2 (10.53%) | 5 (6.85%) | 0.62 | 0.631 |
| BRCA1 | 2 (10.53%) | 5 (6.85%) | 0.62 | 0.631 |
| CIC | 2 (10.53%) | 5 (6.85%) | 0.62 | 0.631 |
| DNMT3B | 2 (10.53%) | 5 (6.85%) | 0.62 | 0.631 |
| ERCC5 | 2 (10.53%) | 5 (6.85%) | 0.62 | 0.631 |
| FGFR1 | 2 (10.53%) | 5 (6.85%) | 0.62 | 0.631 |
| MLH1 | 2 (10.53%) | 5 (6.85%) | 0.62 | 0.631 |
| PIK3CB | 2 (10.53%) | 5 (6.85%) | 0.62 | 0.631 |
| CSDE1 | 2 (11.76%) | 5 (8.06%) | 0.54 | 0.639 |
| NCOA3 | 2 (11.11%) | 6 (8.57%) | 0.37 | 0.664 |
| AR | 2 (10.53%) | 6 (8.22%) | 0.36 | 0.667 |
| ARID2 | 2 (10.53%) | 6 (8.22%) | 0.36 | 0.667 |
| IRS2 | 2 (10.53%) | 6 (8.22%) | 0.36 | 0.667 |
| KIT | 2 (10.53%) | 6 (8.22%) | 0.36 | 0.667 |
| MTOR | 2 (10.53%) | 6 (8.22%) | 0.36 | 0.667 |
| NOTCH3 | 2 (10.53%) | 6 (8.22%) | 0.36 | 0.667 |
| PDGFRA | 2 (10.53%) | 6 (8.22%) | 0.36 | 0.667 |
| PTCH1 | 2 (10.53%) | 6 (8.22%) | 0.36 | 0.667 |
| TSC1 | 2 (10.53%) | 6 (8.22%) | 0.36 | 0.667 |
| PREX2 | 1 (5.88%) | 8 (12.90%) | -1.13 | 0.675 |
| ARID1B | 1 (5.26%) | 8 (10.96%) | -1.06 | 0.679 |
| AXL | 1 (5.26%) | 8 (10.96%) | -1.06 | 0.679 |
| KLF4 | 1 (5.26%) | 8 (10.96%) | -1.06 | 0.679 |
| TERT | 1 (5.26%) | 8 (10.96%) | -1.06 | 0.679 |
| AGO2 | 1 (5.88%) | 9 (14.52%) | -1.3 | 0.681 |
| KMT2A | 1 (5.26%) | 9 (12.33%) | -1.23 | 0.681 |
| MYC | 1 (5.26%) | 9 (12.33%) | -1.23 | 0.681 |
| BRCA2 | 3 (15.79%) | 8 (10.96%) | 0.53 | 0.691 |
| EPHA5 | 3 (15.79%) | 8 (10.96%) | 0.53 | 0.691 |
| NF1 | 3 (15.79%) | 8 (10.96%) | 0.53 | 0.691 |
| POLE | 3 (15.79%) | 8 (10.96%) | 0.53 | 0.691 |
| SMARCA4 | 3 (15.79%) | 9 (12.33%) | 0.36 | 0.707 |
| SOX17 | 3 (15.79%) | 9 (12.33%) | 0.36 | 0.707 |
| APC | 3 (15.79%) | 10 (13.70%) | 0.2 | 0.727 |
| ZFHX3 | 5 (27.78%) | 16 (22.86%) | 0.28 | 0.758 |
| PIK3R1 | 6 (31.58%) | 21 (28.77%) | 0.13 | 0.785 |
| CALR | 0 (0.00%) | 2 (2.86%) | <-10 | 1 |
| CD79A | 0 (0.00%) | 2 (2.86%) | <-10 | 1 |
| FOXO1 | 0 (0.00%) | 2 (2.86%) | <-10 | 1 |
| FYN | 0 (0.00%) | 2 (2.86%) | <-10 | 1 |
| H3C12 | 0 (0.00%) | 2 (2.86%) | <-10 | 1 |
| H3C6 | 0 (0.00%) | 2 (2.86%) | <-10 | 1 |
| INHBA | 0 (0.00%) | 2 (2.86%) | <-10 | 1 |
| NUP93 | 0 (0.00%) | 2 (2.86%) | <-10 | 1 |
| RHEB | 0 (0.00%) | 2 (2.86%) | <-10 | 1 |
| STAT3 | 0 (0.00%) | 2 (2.86%) | <-10 | 1 |
| STAT5A | 0 (0.00%) | 2 (2.86%) | <-10 | 1 |
| PRDM14 | 1 (5.88%) | 6 (9.68%) | -0.72 | 1 |
| DROSHA | 1 (5.88%) | 3 (4.84%) | 0.28 | 1 |
| EPAS1 | 1 (5.88%) | 3 (4.84%) | 0.28 | 1 |
| MSI2 | 1 (5.88%) | 3 (4.84%) | 0.28 | 1 |
| RECQL | 1 (5.88%) | 3 (4.84%) | 0.28 | 1 |
| SLX4 | 1 (5.88%) | 3 (4.84%) | 0.28 | 1 |
| SMYD3 | 1 (5.88%) | 3 (4.84%) | 0.28 | 1 |
| TAP2 | 1 (5.88%) | 3 (4.84%) | 0.28 | 1 |
| CYSLTR2 | 1 (5.88%) | 4 (6.45%) | -0.13 | 1 |
| PRKD1 | 1 (5.88%) | 4 (6.45%) | -0.13 | 1 |
| RTEL1 | 1 (5.88%) | 4 (6.45%) | -0.13 | 1 |
| SOS1 | 1 (5.88%) | 4 (6.45%) | -0.13 | 1 |
| TEK | 1 (5.88%) | 4 (6.45%) | -0.13 | 1 |
| BCL2 | 0 (0.00%) | 2 (2.74%) | <-10 | 1 |
| CBL | 0 (0.00%) | 2 (2.74%) | <-10 | 1 |
| CCND3 | 0 (0.00%) | 2 (2.74%) | <-10 | 1 |
| CDK4 | 0 (0.00%) | 2 (2.74%) | <-10 | 1 |
| CDKN1A | 0 (0.00%) | 2 (2.74%) | <-10 | 1 |
| CDKN2B | 0 (0.00%) | 2 (2.74%) | <-10 | 1 |
| CRLF2 | 0 (0.00%) | 2 (2.74%) | <-10 | 1 |
| EED | 0 (0.00%) | 2 (2.74%) | <-10 | 1 |
| ERCC2 | 0 (0.00%) | 2 (2.74%) | <-10 | 1 |
| GREM1 | 0 (0.00%) | 2 (2.74%) | <-10 | 1 |
| ICOSLG | 0 (0.00%) | 2 (2.74%) | <-10 | 1 |
| IFNGR1 | 0 (0.00%) | 2 (2.74%) | <-10 | 1 |
| IGF2 | 0 (0.00%) | 2 (2.74%) | <-10 | 1 |
| MYCL | 0 (0.00%) | 2 (2.74%) | <-10 | 1 |
| PAK1 | 0 (0.00%) | 2 (2.74%) | <-10 | 1 |
| PDGFRB | 0 (0.00%) | 2 (2.74%) | <-10 | 1 |
| RAD52 | 0 (0.00%) | 2 (2.74%) | <-10 | 1 |
| REL | 0 (0.00%) | 2 (2.74%) | <-10 | 1 |
| SDHB | 0 (0.00%) | 2 (2.74%) | <-10 | 1 |
| SMARCB1 | 0 (0.00%) | 2 (2.74%) | <-10 | 1 |
| SRC | 0 (0.00%) | 2 (2.74%) | <-10 | 1 |
| TNFRSF14 | 0 (0.00%) | 2 (2.74%) | <-10 | 1 |
| YES1 | 0 (0.00%) | 2 (2.74%) | <-10 | 1 |
| MSH3 | 2 (11.76%) | 6 (9.68%) | 0.28 | 1 |
| PTPRD | 2 (10.53%) | 8 (10.96%) | -0.06 | 1 |
| FBXW7 | 3 (15.79%) | 13 (17.81%) | -0.17 | 1 |
| JAK1 | 3 (15.79%) | 13 (17.81%) | -0.17 | 1 |
| BABAM1 | 0 (0.00%) | 3 (4.84%) | <-10 | 1 |
| RRAGC | 0 (0.00%) | 3 (4.84%) | <-10 | 1 |
| WWTR1 | 0 (0.00%) | 3 (4.84%) | <-10 | 1 |
| CARM1 | 0 (0.00%) | 2 (3.23%) | <-10 | 1 |
| CCNQ | 0 (0.00%) | 2 (3.23%) | <-10 | 1 |
| MSI1 | 0 (0.00%) | 2 (3.23%) | <-10 | 1 |
| SESN1 | 0 (0.00%) | 2 (3.23%) | <-10 | 1 |
| TAP1 | 0 (0.00%) | 2 (3.23%) | <-10 | 1 |
| GPS2 | 1 (5.56%) | 3 (4.29%) | 0.37 | 1 |
| PPM1D | 1 (5.56%) | 3 (4.29%) | 0.37 | 1 |
| TRAF2 | 1 (5.56%) | 3 (4.29%) | 0.37 | 1 |
| CXCR4 | 0 (0.00%) | 1 (1.43%) | <-10 | 1 |
| EIF4A2 | 0 (0.00%) | 1 (1.43%) | <-10 | 1 |
| ELOC | 0 (0.00%) | 1 (1.43%) | <-10 | 1 |
| H3-3A | 0 (0.00%) | 1 (1.43%) | <-10 | 1 |
| H3-3B | 0 (0.00%) | 1 (1.43%) | <-10 | 1 |
| H3-4 | 0 (0.00%) | 1 (1.43%) | <-10 | 1 |
| H3C1 | 0 (0.00%) | 1 (1.43%) | <-10 | 1 |
| H3C11 | 0 (0.00%) | 1 (1.43%) | <-10 | 1 |
| ID3 | 0 (0.00%) | 1 (1.43%) | <-10 | 1 |
| MAP3K14 | 0 (0.00%) | 1 (1.43%) | <-10 | 1 |
| NFKBIA | 0 (0.00%) | 1 (1.43%) | <-10 | 1 |
| SRSF2 | 0 (0.00%) | 1 (1.43%) | <-10 | 1 |
| AKT2 | 1 (5.26%) | 3 (4.11%) | 0.36 | 1 |
| AMER1 | 1 (5.26%) | 3 (4.11%) | 0.36 | 1 |
| ASXL2 | 1 (5.26%) | 3 (4.11%) | 0.36 | 1 |
| BARD1 | 1 (5.26%) | 3 (4.11%) | 0.36 | 1 |
| EGFR | 1 (5.26%) | 3 (4.11%) | 0.36 | 1 |
| ERG | 1 (5.26%) | 3 (4.11%) | 0.36 | 1 |
| FGF3 | 1 (5.26%) | 3 (4.11%) | 0.36 | 1 |
| FGF4 | 1 (5.26%) | 3 (4.11%) | 0.36 | 1 |
| FLT1 | 1 (5.26%) | 3 (4.11%) | 0.36 | 1 |
| IGF1R | 1 (5.26%) | 3 (4.11%) | 0.36 | 1 |
| IL7R | 1 (5.26%) | 3 (4.11%) | 0.36 | 1 |
| MYOD1 | 1 (5.26%) | 3 (4.11%) | 0.36 | 1 |
| NOTCH4 | 1 (5.26%) | 3 (4.11%) | 0.36 | 1 |
| PNRC1 | 1 (5.26%) | 3 (4.11%) | 0.36 | 1 |
| RIT1 | 1 (5.26%) | 3 (4.11%) | 0.36 | 1 |
| RPS6KB2 | 1 (5.26%) | 3 (4.11%) | 0.36 | 1 |
| SMAD3 | 1 (5.26%) | 3 (4.11%) | 0.36 | 1 |
| SMARCD1 | 1 (5.26%) | 3 (4.11%) | 0.36 | 1 |
| SOX9 | 1 (5.26%) | 3 (4.11%) | 0.36 | 1 |
| STK40 | 1 (5.26%) | 3 (4.11%) | 0.36 | 1 |
| TET2 | 1 (5.26%) | 3 (4.11%) | 0.36 | 1 |
| ZRSR2 | 2 (11.11%) | 7 (10.00%) | 0.15 | 1 |
| MGA | 2 (11.11%) | 8 (11.43%) | -0.04 | 1 |
| KMT5A | 0 (0.00%) | 1 (1.61%) | <-10 | 1 |
| KNSTRN | 0 (0.00%) | 1 (1.61%) | <-10 | 1 |
| NTHL1 | 0 (0.00%) | 1 (1.61%) | <-10 | 1 |
| RAC2 | 0 (0.00%) | 1 (1.61%) | <-10 | 1 |
| BCOR | 1 (5.26%) | 7 (9.59%) | -0.87 | 1 |
| CDK12 | 1 (5.26%) | 7 (9.59%) | -0.87 | 1 |
| DNMT3A | 1 (5.26%) | 7 (9.59%) | -0.87 | 1 |
| NFE2L2 | 1 (5.26%) | 7 (9.59%) | -0.87 | 1 |
| RICTOR | 1 (5.26%) | 7 (9.59%) | -0.87 | 1 |
| KMT2B | 4 (23.53%) | 16 (25.81%) | -0.13 | 1 |
| AKT1 | 0 (0.00%) | 3 (4.11%) | <-10 | 1 |
| AURKB | 0 (0.00%) | 3 (4.11%) | <-10 | 1 |
| BCL2L1 | 0 (0.00%) | 3 (4.11%) | <-10 | 1 |
| BCL2L11 | 0 (0.00%) | 3 (4.11%) | <-10 | 1 |
| CDC73 | 0 (0.00%) | 3 (4.11%) | <-10 | 1 |
| CDK8 | 0 (0.00%) | 3 (4.11%) | <-10 | 1 |
| ERCC4 | 0 (0.00%) | 3 (4.11%) | <-10 | 1 |
| FGFR4 | 0 (0.00%) | 3 (4.11%) | <-10 | 1 |
| GNA11 | 0 (0.00%) | 3 (4.11%) | <-10 | 1 |
| HRAS | 0 (0.00%) | 3 (4.11%) | <-10 | 1 |
| IDH2 | 0 (0.00%) | 3 (4.11%) | <-10 | 1 |
| IGF1 | 0 (0.00%) | 3 (4.11%) | <-10 | 1 |
| LATS2 | 0 (0.00%) | 3 (4.11%) | <-10 | 1 |
| MAP2K4 | 0 (0.00%) | 3 (4.11%) | <-10 | 1 |
| MAPK1 | 0 (0.00%) | 3 (4.11%) | <-10 | 1 |
| MDC1 | 0 (0.00%) | 3 (4.11%) | <-10 | 1 |
| MET | 0 (0.00%) | 3 (4.11%) | <-10 | 1 |
| NF2 | 0 (0.00%) | 3 (4.11%) | <-10 | 1 |
| PIK3C3 | 0 (0.00%) | 3 (4.11%) | <-10 | 1 |
| PMS1 | 0 (0.00%) | 3 (4.11%) | <-10 | 1 |
| RAD51C | 0 (0.00%) | 3 (4.11%) | <-10 | 1 |
| SHQ1 | 0 (0.00%) | 3 (4.11%) | <-10 | 1 |
| SMAD4 | 0 (0.00%) | 3 (4.11%) | <-10 | 1 |
| TBX3 | 0 (0.00%) | 3 (4.11%) | <-10 | 1 |
| TENT5C | 0 (0.00%) | 3 (4.11%) | <-10 | 1 |
| WT1 | 0 (0.00%) | 3 (4.11%) | <-10 | 1 |
| XIAP | 0 (0.00%) | 3 (4.11%) | <-10 | 1 |
| GLI1 | 1 (5.56%) | 6 (8.57%) | -0.63 | 1 |
| CSF3R | 0 (0.00%) | 3 (4.29%) | <-10 | 1 |
| HLA-A | 0 (0.00%) | 3 (4.29%) | <-10 | 1 |
| INHA | 0 (0.00%) | 3 (4.29%) | <-10 | 1 |
| MALT1 | 0 (0.00%) | 3 (4.29%) | <-10 | 1 |
| MST1 | 0 (0.00%) | 3 (4.29%) | <-10 | 1 |
| RAD21 | 0 (0.00%) | 3 (4.29%) | <-10 | 1 |
| ABRAXAS1 | 0 (0.00%) | 1 (1.37%) | <-10 | 1 |
| AKAP8 | 0 (0.00%) | 1 (1.37%) | <-10 | 1 |
| BBC3 | 0 (0.00%) | 1 (1.37%) | <-10 | 1 |
| BSN | 0 (0.00%) | 1 (1.37%) | <-10 | 1 |
| CAMTA1 | 0 (0.00%) | 1 (1.37%) | <-10 | 1 |
| CCND2 | 0 (0.00%) | 1 (1.37%) | <-10 | 1 |
| CD274 | 0 (0.00%) | 1 (1.37%) | <-10 | 1 |
| CD276 | 0 (0.00%) | 1 (1.37%) | <-10 | 1 |
| CDKN1B | 0 (0.00%) | 1 (1.37%) | <-10 | 1 |
| EPCAM | 0 (0.00%) | 1 (1.37%) | <-10 | 1 |
| EWSR1 | 0 (0.00%) | 1 (1.37%) | <-10 | 1 |
| FAM193A | 0 (0.00%) | 1 (1.37%) | <-10 | 1 |
| FOXL2 | 0 (0.00%) | 1 (1.37%) | <-10 | 1 |
| FUBP1 | 0 (0.00%) | 1 (1.37%) | <-10 | 1 |
| GATA1 | 0 (0.00%) | 1 (1.37%) | <-10 | 1 |
| GON4L | 0 (0.00%) | 1 (1.37%) | <-10 | 1 |
| GSE1 | 0 (0.00%) | 1 (1.37%) | <-10 | 1 |
| IKBKE | 0 (0.00%) | 1 (1.37%) | <-10 | 1 |
| IL10 | 0 (0.00%) | 1 (1.37%) | <-10 | 1 |
| IPO11 | 0 (0.00%) | 1 (1.37%) | <-10 | 1 |
| LMO1 | 0 (0.00%) | 1 (1.37%) | <-10 | 1 |
| LRRC20 | 0 (0.00%) | 1 (1.37%) | <-10 | 1 |
| MAGT1 | 0 (0.00%) | 1 (1.37%) | <-10 | 1 |
| MAP2K1 | 0 (0.00%) | 1 (1.37%) | <-10 | 1 |
| MAX | 0 (0.00%) | 1 (1.37%) | <-10 | 1 |
| MC4R | 0 (0.00%) | 1 (1.37%) | <-10 | 1 |
| MCL1 | 0 (0.00%) | 1 (1.37%) | <-10 | 1 |
| MDM4 | 0 (0.00%) | 1 (1.37%) | <-10 | 1 |
| MRPS18B | 0 (0.00%) | 1 (1.37%) | <-10 | 1 |
| MTREX | 0 (0.00%) | 1 (1.37%) | <-10 | 1 |
| NANOS2 | 0 (0.00%) | 1 (1.37%) | <-10 | 1 |
| NKX2-1 | 0 (0.00%) | 1 (1.37%) | <-10 | 1 |
| NPAS2 | 0 (0.00%) | 1 (1.37%) | <-10 | 1 |
| NPM1 | 0 (0.00%) | 1 (1.37%) | <-10 | 1 |
| PDGFB | 0 (0.00%) | 1 (1.37%) | <-10 | 1 |
| PHOX2B | 0 (0.00%) | 1 (1.37%) | <-10 | 1 |
| PIK3R3 | 0 (0.00%) | 1 (1.37%) | <-10 | 1 |
| PRKAR1A | 0 (0.00%) | 1 (1.37%) | <-10 | 1 |
| RAD51 | 0 (0.00%) | 1 (1.37%) | <-10 | 1 |
| RAD51B | 0 (0.00%) | 1 (1.37%) | <-10 | 1 |
| RAD51D | 0 (0.00%) | 1 (1.37%) | <-10 | 1 |
| RARA | 0 (0.00%) | 1 (1.37%) | <-10 | 1 |
| RHOA | 0 (0.00%) | 1 (1.37%) | <-10 | 1 |
| SDHC | 0 (0.00%) | 1 (1.37%) | <-10 | 1 |
| SOCS1 | 0 (0.00%) | 1 (1.37%) | <-10 | 1 |
| SPATA31F2P | 0 (0.00%) | 1 (1.37%) | <-10 | 1 |
| SUFU | 0 (0.00%) | 1 (1.37%) | <-10 | 1 |
| TMEM127 | 0 (0.00%) | 1 (1.37%) | <-10 | 1 |
| TRAF7 | 0 (0.00%) | 1 (1.37%) | <-10 | 1 |
| UNC13A | 0 (0.00%) | 1 (1.37%) | <-10 | 1 |
| VTCN1 | 0 (0.00%) | 1 (1.37%) | <-10 | 1 |
| ALK | 1 (5.26%) | 6 (8.22%) | -0.64 | 1 |
| CARD11 | 1 (5.26%) | 6 (8.22%) | -0.64 | 1 |
| KDM5C | 1 (5.26%) | 6 (8.22%) | -0.64 | 1 |
| NCOR1 | 1 (5.26%) | 6 (8.22%) | -0.64 | 1 |
| RB1 | 1 (5.26%) | 6 (8.22%) | -0.64 | 1 |
| RECQL4 | 1 (5.26%) | 6 (8.22%) | -0.64 | 1 |
| ROS1 | 1 (5.26%) | 6 (8.22%) | -0.64 | 1 |
| SMO | 1 (5.26%) | 6 (8.22%) | -0.64 | 1 |
| BIRC3 | 1 (5.56%) | 4 (5.71%) | -0.04 | 1 |
| ARAF | 1 (5.26%) | 5 (6.85%) | -0.38 | 1 |
| DOT1L | 1 (5.26%) | 5 (6.85%) | -0.38 | 1 |
| FGFR3 | 1 (5.26%) | 5 (6.85%) | -0.38 | 1 |
| FLT3 | 1 (5.26%) | 5 (6.85%) | -0.38 | 1 |
| FOXA1 | 1 (5.26%) | 5 (6.85%) | -0.38 | 1 |
| KDM6A | 1 (5.26%) | 5 (6.85%) | -0.38 | 1 |
| MAP2K2 | 1 (5.26%) | 5 (6.85%) | -0.38 | 1 |
| MEN1 | 1 (5.26%) | 5 (6.85%) | -0.38 | 1 |
| PBRM1 | 1 (5.26%) | 5 (6.85%) | -0.38 | 1 |
| PIK3C2G | 1 (5.26%) | 5 (6.85%) | -0.38 | 1 |
| PLK2 | 1 (5.26%) | 5 (6.85%) | -0.38 | 1 |
| PTPN11 | 1 (5.26%) | 5 (6.85%) | -0.38 | 1 |
| RPTOR | 1 (5.26%) | 5 (6.85%) | -0.38 | 1 |
| TOP1 | 1 (5.26%) | 5 (6.85%) | -0.38 | 1 |
| ASXL1 | 2 (10.53%) | 7 (9.59%) | 0.13 | 1 |
| CASP8 | 2 (10.53%) | 7 (9.59%) | 0.13 | 1 |
| CCND1 | 2 (10.53%) | 7 (9.59%) | 0.13 | 1 |
| DNMT1 | 2 (10.53%) | 7 (9.59%) | 0.13 | 1 |
| EPHA3 | 2 (10.53%) | 7 (9.59%) | 0.13 | 1 |
| RASA1 | 2 (10.53%) | 7 (9.59%) | 0.13 | 1 |
| RUNX1 | 2 (10.53%) | 7 (9.59%) | 0.13 | 1 |
| TSC2 | 2 (10.53%) | 7 (9.59%) | 0.13 | 1 |
| ERBB2 | 2 (10.53%) | 10 (13.70%) | -0.38 | 1 |
| INSR | 2 (10.53%) | 10 (13.70%) | -0.38 | 1 |
| BRAF | 1 (5.26%) | 4 (5.48%) | -0.06 | 1 |
| BRIP1 | 1 (5.26%) | 4 (5.48%) | -0.06 | 1 |
| DAXX | 1 (5.26%) | 4 (5.48%) | -0.06 | 1 |
| DDR2 | 1 (5.26%) | 4 (5.48%) | -0.06 | 1 |
| ERBB4 | 1 (5.26%) | 4 (5.48%) | -0.06 | 1 |
| ETV1 | 1 (5.26%) | 4 (5.48%) | -0.06 | 1 |
| FH | 1 (5.26%) | 4 (5.48%) | -0.06 | 1 |
| GATA3 | 1 (5.26%) | 4 (5.48%) | -0.06 | 1 |
| INPP4A | 1 (5.26%) | 4 (5.48%) | -0.06 | 1 |
| JAK2 | 1 (5.26%) | 4 (5.48%) | -0.06 | 1 |
| JAK3 | 1 (5.26%) | 4 (5.48%) | -0.06 | 1 |
| MAP3K13 | 1 (5.26%) | 4 (5.48%) | -0.06 | 1 |
| MEF2B | 1 (5.26%) | 4 (5.48%) | -0.06 | 1 |
| PIK3CD | 1 (5.26%) | 4 (5.48%) | -0.06 | 1 |
| PIK3CG | 1 (5.26%) | 4 (5.48%) | -0.06 | 1 |
| RAD50 | 1 (5.26%) | 4 (5.48%) | -0.06 | 1 |
| RET | 1 (5.26%) | 4 (5.48%) | -0.06 | 1 |
| TMPRSS2 | 1 (5.26%) | 4 (5.48%) | -0.06 | 1 |
| U2AF1 | 1 (5.26%) | 4 (5.48%) | -0.06 | 1 |
| MST1R | 1 (5.56%) | 5 (7.14%) | -0.36 | 1 |
| PGR | 1 (5.56%) | 5 (7.14%) | -0.36 | 1 |
| TCF7L2 | 1 (5.56%) | 5 (7.14%) | -0.36 | 1 |

| Table S3. Tier I and Tier II mutation genes in EC-3rd-SYSU cohort | | | | | |
| --- | --- | --- | --- | --- | --- |
|  | Young group (count) | Young group (ratio) | Old group (count) | Old group (ratio) | Log2 Ratio  (young vs old) |
| PTEN | 8 | 80.00% | 8 | 100.00% | 0 |
| ARID1A | 5 | 50.00% | 7 | 87.50% | -0.485 |
| PIK3CA | 7 | 70.00% | 6 | 75.00% | 0.222 |
| KRAS | 2 | 20.00% | 4 | 50.00% | -1 |
| MSH6 | 0 | 0.00% | 3 | 37.50% | <-10 |
| RNF43 | 0 | 0.00% | 3 | 37.50% | <-10 |
| TP53 | 1 | 10.00% | 2 | 25.00% | -1 |
| POLE | 0 | 0.00% | 2 | 25.00% | <-10 |
| SMARCA4 | 0 | 0.00% | 2 | 25.00% | <-10 |
| KMT2D | 0 | 0.00% | 2 | 25.00% | <-10 |
| FGFR2 | 3 | 30.00% | 1 | 12.50% | 1.585 |
| EZH2 | 1 | 10.00% | 1 | 12.50% | 0 |
| PIK3R1 | 1 | 10.00% | 1 | 12.50% | 0 |
| PBRM1 | 0 | 0.00% | 1 | 12.50% | <-10 |
| ESR1 | 0 | 0.00% | 1 | 12.50% | <-10 |
| FH | 0 | 0.00% | 1 | 12.50% | <-10 |
| JAK1 | 0 | 0.00% | 1 | 12.50% | <-10 |
| ATM | 0 | 0.00% | 1 | 12.50% | <-10 |
| ARID2 | 0 | 0.00% | 1 | 12.50% | <-10 |
| MSH3 | 0 | 0.00% | 1 | 12.50% | <-10 |
| MLH3 | 0 | 0.00% | 1 | 12.50% | <-10 |
| ARID1B | 0 | 0.00% | 1 | 12.50% | <-10 |
| FANCM | 0 | 0.00% | 1 | 12.50% | <-10 |
| FANCE | 0 | 0.00% | 1 | 12.50% | <-10 |
| DICER1 | 0 | 0.00% | 1 | 12.50% | <-10 |
| BLM | 0 | 0.00% | 1 | 12.50% | <-10 |
| BRCA1 | 0 | 0.00% | 1 | 12.50% | <-10 |
| NFE2L2 | 0 | 0.00% | 1 | 12.50% | <-10 |
| ERBB2 | 0 | 0.00% | 1 | 12.50% | <-10 |
| CDKN2A | 0 | 0.00% | 1 | 12.50% | <-10 |
| JAK1 | 0 | 0.00% | 1 | 12.50% | <-10 |
| APC | 0 | 0.00% | 1 | 12.50% | <-10 |
| ATR | 0 | 0.00% | 1 | 12.50% | <-10 |
| RAD50 | 0 | 0.00% | 1 | 12.50% | <-10 |
| RB1 | 0 | 0.00% | 1 | 12.50% | <-10 |
| CTNNB1 | 3 | 30.00% | 0 | 0.00% | >10 |
| ERBB3 | 2 | 20.00% | 0 | 0.00% | >10 |
| MAX | 1 | 10.00% | 0 | 0.00% | >10 |
| CTCF | 1 | 10.00% | 0 | 0.00% | >10 |
| TSC1 | 1 | 10.00% | 0 | 0.00% | >10 |
| FANCC | 1 | 10.00% | 0 | 0.00% | >10 |
| MUTYH | 1 | 10.00% | 0 | 0.00% | >10 |
| NF1 | 1 | 10.00% | 0 | 0.00% | >10 |
| PPP2R1A | 1 | 10.00% | 0 | 0.00% | >10 |
| PMS1 | 1 | 10.00% | 0 | 0.00% | >10 |
| MSH2 | 1 | 10.00% | 0 | 0.00% | >10 |
| ATR | 1 | 10.00% | 0 | 0.00% | >10 |
| FBXW7 | 1 | 10.00% | 0 | 0.00% | >10 |
